# Temporal Trends in Six Major Cancers: Implications for Lifestyle-Based Cancer Prevention and Early Detection Across the Life Course

**DOI:** 10.3390/cancers18193149

**Published:** 2026-09-29

**Authors:** Itunu Sokale, Omar Rosales, Hashem El-Serag, Natalia Khalaf, Aaron P. Thrift, Victor Adekanmbi, Abiodun Oluyomi

**Affiliations:** 1 Section of Epidemiology and Population Sciences, Department of Medicine, Baylor College of Medicine, Houston, TX 77030, USA; 2 Dan L Duncan Comprehensive Cancer Center, Baylor College of Medicine, Houston, TX 77030, USA; 3 Section of Gastroenterology and Hepatology, Department of Medicine, Baylor College of Medicine, Houston, TX 77030, USA; 4 Center for Innovations in Quality, Effectiveness and Safety (IQuESt), Michael E. DeBakey Veterans Affairs Medical Center and Baylor College of Medicine, Houston, TX 77030, USA; 5 Texas Medical Center Digestive Disease Center, Houston, TX 77030, USA; 6 Department of Obstetrics and Gynecology, The University of Texas Medical Branch, Galveston, TX 77555, USA

**Keywords:** cancer trends, cancer incidence, age at onset, stage at diagnosis, breast, colorectal, liver, lung, pancreas, prostate

## Abstract

Background/Objectives: Cancer remains a serious public health challenge in the United States. Six major cancers (female breast, colorectal, liver, lung/bronchus, pancreas, and prostate) account for a substantial proportion of the national cancer burden. Understanding the changes in the incidence of these cancers over time can provide insights for prevention and control. This study examined temporal trends in the cancer incidence according to age at onset and stage at diagnosis. Methods: This population-based study analyzed data from the United States Cancer Statistics database for 2001–2023, including all 50 states and the District of Columbia. Joinpoint regression was used to estimate annual percent changes (APCs) and average annual percent changes (AAPCs), with corresponding 95% confidence intervals (CIs), based on age-adjusted incidence rates per 100,000 people. Of note, incidence rates from 2020 data were excluded from the Joinpoint models because COVID-19 pandemic disruptions affected cancer screening, diagnosis, and reporting. Results: From 2001 to 2023, the incidence of lung and colorectal cancers declined, particularly among older adults (aged ≥50 years) (lung cancer: AAPC −2.09; 95% CI: −2.27, −1.90; colorectal cancer AAPC −2.70; 95% CI: −2.83, −2.56). In contrast, the incidence of colorectal, pancreatic, and breast cancers increased among younger adults (aged <50 years), whereas the incidence of liver and lung cancer declined. Moreover, prostate cancer incidence declined over time despite modest rebound in recent years (AAPC 1.83; 95% CI: 0.53, 3.14). Stage-specific analyses revealed increasing rates of early-stage diagnoses for several cancers, while late-stage cancer incidence increased for pancreatic and colorectal cancers (especially among younger adults). Conclusions: Cancer incidence varied substantially by cancer type, age, and stage at diagnosis. The rising incidence of colorectal, pancreatic, and breast cancers among younger adults underscores the need for continued research into risk factors, early detection, and prevention strategies.

## Simple Summary

Six major cancers (female breast, colorectal, liver, lung, pancreas, and prostate) substantially impact public health in the U.S. because together they account for a large portion of cancer burden. Monitoring incidence trends can help inform effective prevention strategies. While lung and colorectal cancers’ incidence rates declined overall, rates of colorectal, pancreatic, and breast cancers increased significantly among younger adults. In contrast, liver, lung, and prostate cancers decreased among younger adults. There has been a general decline in late-stage cancer diagnoses, although lung and pancreatic cancers remain predominantly diagnosed in late stages. These trends underscore the unequal progress and ongoing challenges in cancer prevention, particularly among younger adults.

## 1. Introduction

Cancer remains a serious public health issue in the United States (U.S.). Approximately 2 million individuals were diagnosed with new cases of cancer and over 600,000 cancer-related deaths occurred in 2025 [1]. Cancers with substantial public health impact in the U.S. include lung/bronchial, colorectal, pancreatic, breast, prostate, and liver cancers. Together, these six cancers account for a large proportion of cancer incidence, healthcare utilization, mortality, and survivorship in the U.S. [2,3].

Many of these cancers are partially preventable if modifiable risk factors such as tobacco use, alcohol consumption, obesity, or certain infections (e.g., viral hepatitis) are avoided [4]. Although prior studies suggest that rates of several leading cancers have declined in recent decades, evidence indicates that improvements are not uniform across age groups [5,6]. Cancer is primarily a disease of aging; however, recent studies have shown a worldwide increase in incidence among individuals under 50 years of age [7,8,9]. Early-onset cancers can be defined as cancers that usually occur later in life but are diagnosed in adults <50 years of age, and late-onset cancers are those diagnosed among adults ≥50 years of age [10]. While the drivers of this concerning early-onset cancer trend remain poorly understood, it is essential to monitor temporal patterns in major cancers across age groups to gain insights into changes in their burden over time.

Early detection is crucial because stage at diagnosis is an important indicator of cancer control practices. Cancer stage at diagnosis influences treatment opportunities and is strongly linked with survival outcomes [11,12,13,14], yet nearly half of cancers are diagnosed at an advanced stage [14]. Although temporal trends in individual cancers have been extensively reported, comprehensive analyses comparing major cancers simultaneously by age group and stage at diagnosis using data through 2023, covering all U.S. 50 states and Washington, DC, remain scarce. Such studies may provide important data for developing strategic interventions for cancer risk reduction, screening guidelines, and early detection efforts. The insights can help identify subpopulations that may benefit from additional interventions. Therefore, the primary aim of this study was to evaluate temporal trends in the incidence of six major cancers in the U.S. and compare patterns between early-onset and late-onset disease. The secondary objectives were to identify age-specific contributions to these trends using narrower age categories and to explore temporal changes in stage at diagnosis across cancer types.

## 2. Materials and Methods

In this population-based study, we explored the temporal trends in age-adjusted incidence rates of six leading cancers in the United States.

### 2.1. Data Source, Collection, and Retrieval

We analyzed cancer incidence data from the United States Cancer Statistics (USCS) public use database, covering all 50 states and the District of Columbia. The USCS is a comprehensive population-based cancer surveillance system that combines data from the Centers for Disease Control and Prevention’s (CDC’s) National Program of Cancer Registries (NPCR) and the National Cancer Institute’s (NCI’s) Surveillance, Epidemiology, and End Results (SEER) databases [15]. Data on all newly diagnosed cancer cases from patient records at medical facilities, including hospitals, physician offices, surgical centers, and pathology laboratories, are reported to NPCR- and SEER-supported central cancer registries [15]. The central cancer registries also conduct active surveillance for state vital records to identify and document cancer-related deaths that may not have been reported as cancer cases; hence, USCS data include cases ascertained through death certificates and autopsies. To ensure consistency and comparability, the registries compile the data using standardized data elements and coding systems established by the North American Association of Central Cancer Registries (NAACCR). The registries ensure data meet the high-quality standards specified by the USCS publication standards [16], and submit data annually to CDC and NCI, which are then combined into a single dataset, that includes information on cancer incidence, population, demographics, and tumor characteristics. We retrieved the USCS public use data (June 2026 release) for 2001 (earliest available data) to 2023 using the NCI SEER*Stat software version 9.0.43.0 (released on 27 March 2026) [17]. The June 2026 Release data covered 99.1% of the U.S. population as of the date of access in August 2026 [15]. Our study was exempt from ethical review and informed consent procedures because the analyzed data were de-identified and publicly available. We adhered to the Strengthening the Reporting of Observational Studies in Epidemiology (STROBE) reporting guidelines [18].

### 2.2. Case Definition

We obtained data on new cases of primary malignant (female breast, colorectal, liver, lung and bronchus, pancreatic, and prostate) cancers diagnosed among U.S. adults aged 20 years or older, from 1 January 2001 to 31 December 2023. The outcome of interest was the incidence of first primary malignant cancers. To avoid counting multiple cancers occurring in the same individual, analyses were restricted to first primary malignant cancers. Cases were identified using the Sequence Number Central variable in the USCS database and included cancers coded as “one primary only” or first of two or more primaries. Thus, each individual contributed only one cancer diagnosis to the analysis. Primary site and histology codes were selected according to the International Classification of Diseases for Oncology, Third Edition [ICD-O-3]/WHO 2008 Definition [19]. The specific codes used in the selection were female breast (C500–C509); colon and rectum (C180, C182–C189, C199, C209, and C260); liver (C220); lung/bronchus (C340–C349); pancreas (C250–C259); and prostate (C619). Male breast cancer cases were excluded from the analysis because their small sample size limited the statistical power to generate reliable results. Furthermore, appendiceal malignancies were excluded from this study because they are considered distinct from malignancies arising from the colon and rectum. Five cases (colorectal (two cases) and prostate (three)) were excluded because of discrepancies in histologic behavior and summary stages. Data included all racial/ethnic groups: Hispanic, non-Hispanic American Indian/Alaska Native, non-Hispanic Asian/Pacific Islanders, non-Hispanic Black, and non-Hispanic White.

### 2.3. Study Variables

Cancer case counts and incidence rates were retrieved from the USCS database using the SEER*Stat (version 9.0.43.0), by sex (female and male), age, and stage at diagnosis. For the primary analyses, we combined the USCS-provided 5-year age categories into 20–49 years (early-onset) and ≥50 years (late-onset) of age. For the secondary analyses, we used 20–39, 40–49, 50–59, 60–69, and ≥70 years of age. We operationalized stage at diagnosis using the USCS-provided Merged Summary Stage variable, a standardized variable that accounts for staging across time periods with different staging classifications. The Merged Summary Stage variable is created from the SEER Summary Stage 2000 (for cases diagnosed in 2001–2003, 2016, or 2017), Derived SEER Summary Stage 2000 (cases diagnosed from 2004–2015), and Summary Stage 2018 (cases diagnosed in 2018 and later) [20]. Within Seer*Stat, we used the Merged Summary Stage variable to classify stage at diagnosis as (1) early (localized); (2) late (regional by direct extension only, regional lymph nodes only, regional by both, regional not otherwise specified, or distant metastasis); or (3) unstaged (unknown or unstaged, not applicable) cancer.

### 2.4. Statistical Analysis

We defined incidence rates as the annual number of individuals diagnosed with cancer (numerator) per 100,000 people (denominator). For female breast and prostate cancers, the denominators were 100,000 women and 100,000 men, respectively. Using SEER*Stat, we calculated age-adjusted incidence rates (AAIRs) per 100,000 people in the corresponding population, adjusted to the 2000 U.S. standard population, which is the official population available through the USCS database at the time of this study. AAIRs are weighted averages of age-specific crude cancer incidence rates, weighted by the proportions of persons in the corresponding age groups in the U.S. standard population. AAIRs eliminate the confounding effect of age distributions on cancer incidence. We exported AAIRs for each cancer type, overall and stratified by sex (for cancers occurring in both men and women), age, and stage at diagnosis to the NCI Joinpoint Regression Program (version 6.1.0) [21]. We utilized joinpoint regression models with a log-linear specification to analyze temporal trends in AAIRs and calculated the annual percent changes (APCs) and average annual percent changes (AAPCs). In the log-linear joinpoint model, the slope of each trend segment represents the rate of change in the natural logarithm of the AAIR over time. Each slope coefficient is transformed into an APC, which quantifies the average yearly percent increase or decrease in incidence during a specific segment.

Positive and negative APCs or AAPCs indicate increasing and decreasing trends, respectively, while statistical significance is determined based on the corresponding confidence intervals and hypothesis tests. Following the CDC/NCI recommendations for cancer incidence trend analyses in the post-COVID-19 era, we excluded 2020 incidence rates from Joinpoint models [22,23]. The 2020 data point is considered an outlier reflecting temporal disruptions in cancer screening, diagnosis, and reporting rather than underlying changes in cancer incidence [23,24,25]. We used a new feature in the updated NCI Joinpoint program that allows data points to be excluded from joinpoint models, so we excluded the 2020 data point [23]. Thus, the omitted 2020 data point was treated as a missing observation in the models and was not used in estimating joinpoints, APCs, or AAPCs. Joinpoints identify statistically significant slope changes, indicating time points at which the direction or magnitude of the temporal trend changed. AAPCs summarize the overall trend across the entire study period as weighted averages of segment-specific APCs. We allowed a minimum of 0 and a maximum of three joinpoints, with a minimum of four observations between two joinpoints and between a joinpoint and either end of the study period to prevent unstable short-lived trend segments [26,27,28]. Model selection was based on the Monte Carlo permutation test using a significance level of 0.05 (two-sided) for all tests [29]. An error model incorporating first-order autocorrelation, estimated from the data, was used to account for serial correlation between annual rates. APC, AAPC, and Tau confidence intervals were estimated using the parametric method. Corresponding 95% confidence intervals (CIs) were generated for APC and AAPC estimates to assess statistical significance. We present gender-, age group-, and stage-specific estimates for each cancer type.

## 3. Results

Table 1 presents annual case counts, population denominators, AAIRs, and changes in incidence from 2001 to 2023. Cancer incidence trends varied considerably by cancer type, age at onset, and stage at diagnosis. Detailed age- and stage-specific results are presented in Tables S1–S12.

### 3.1. Female Breast Cancer Incidence and Trends

Between 2001 and 2023, approximately 4,431,459 women aged ≥20 years were diagnosed with invasive breast cancer (Table 2). Most cases were late-onset (78.54%), with less than one-fifth (21.46%) being early-onset (Table 3 and Table S2). The AAIR for female breast cancer was 149.25 (95% CI: 149.11, 149.39) per 100,000 women (Table 2).

**Figure 1:**
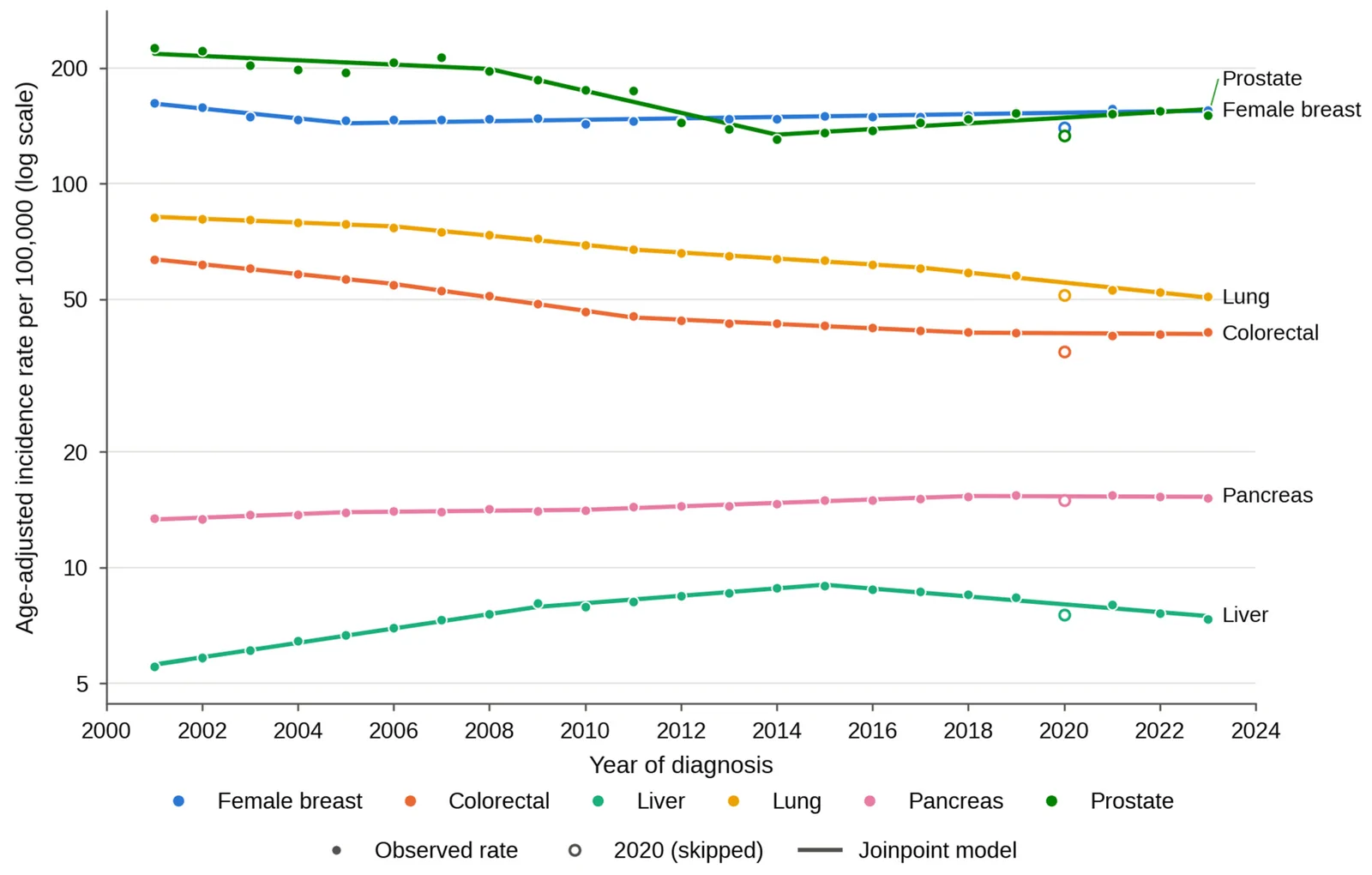
Trends in age-adjusted incidence rates of six major cancers among individuals aged ≥20 years in the United States from 2001 to 2023. Footnotes: Due to disruptions in cancer diagnosis and reporting, 2020 data point was excluded from the trend analysis.

**Figure 2:**
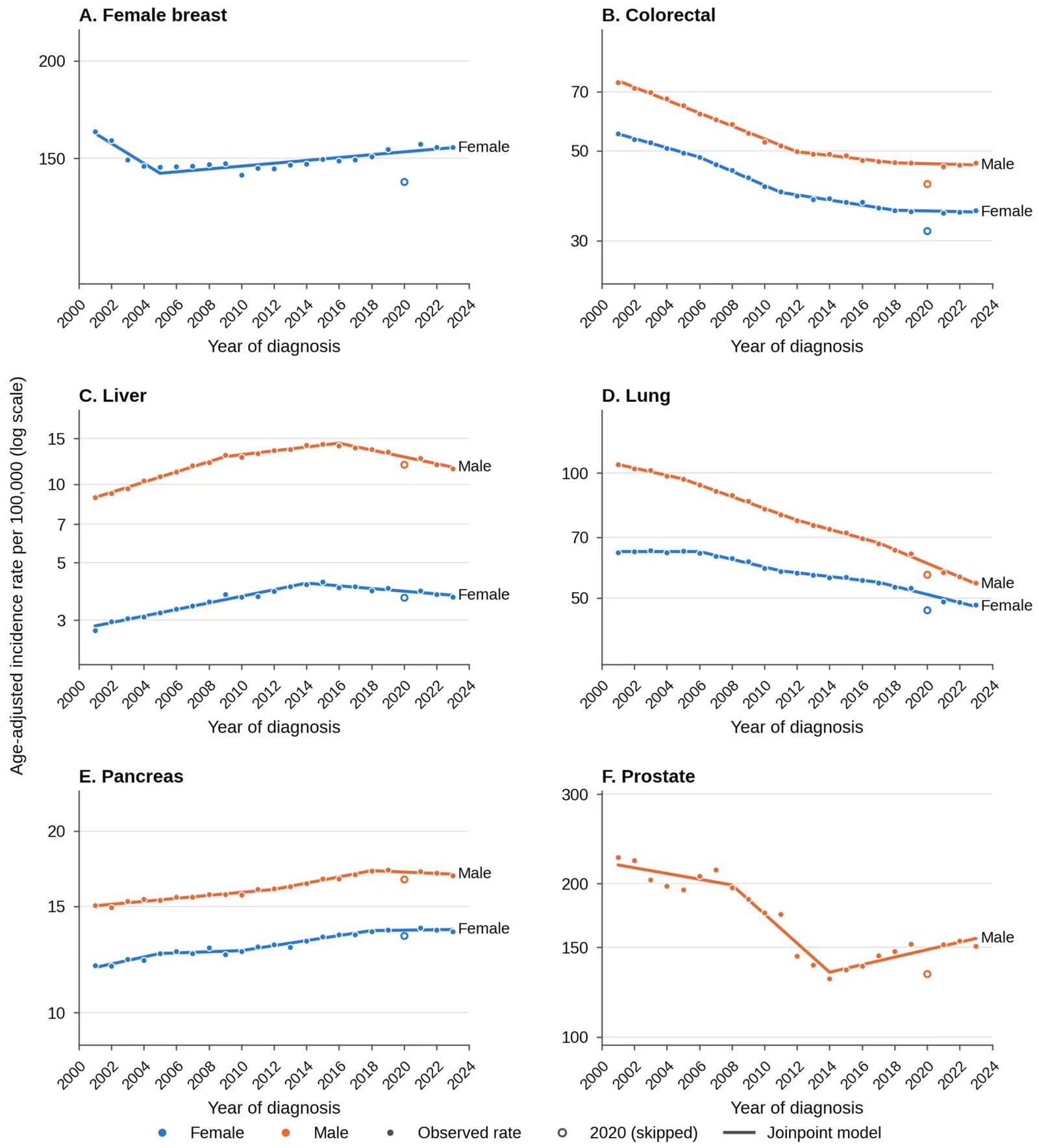
Trends in age-adjusted incidence rates of six major cancers among individuals aged ≥20 years in the United States from 2001 to 2023 by sex. Footnotes: Due to disruptions in cancer diagnosis and reporting, 2020 data point was excluded from the trend analysis.

**Figure 3:**
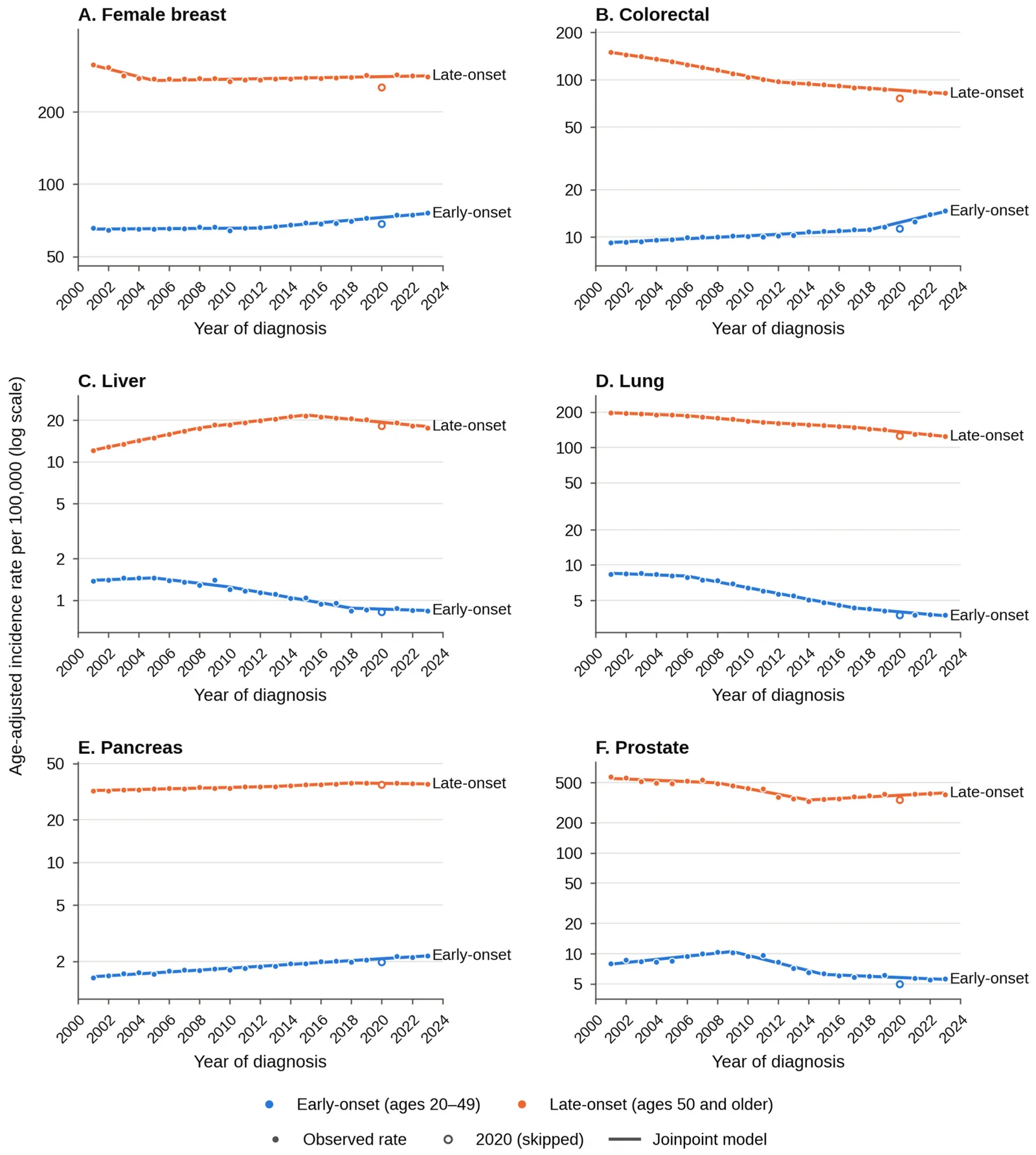
Trends in age-adjusted incidence rates of six major cancers among women aged ≥20 years in the United States from 2001 to 2023 by age of onset. Footnotes: Due to disruptions in cancer diagnosis and reporting, 2020 data point was excluded from the trend analysis.

**Figure 4:**
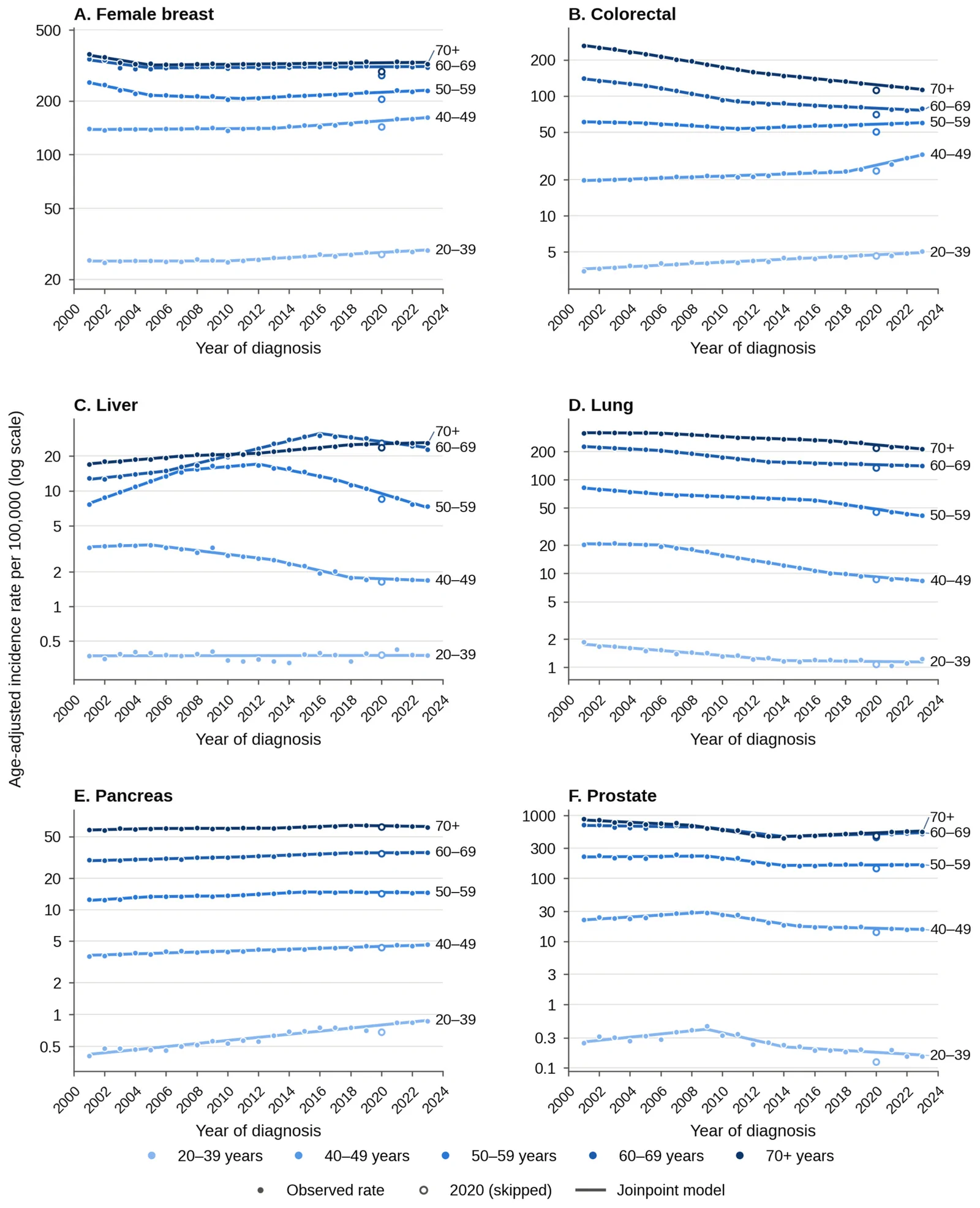
Trends in age-adjusted incidence rates of six major cancers among individuals aged ≥20 years in the United States from 2001 to 2023 by age categories. Footnotes: Due to disruptions in cancer diagnosis and reporting, 2020 data point was excluded from the trend analysis.

Overall incidence (Figure 1 and Figure 2) showed no statistically significant annual change during the study period (AAPC −0.19; 95% CI: −0.43, 0.05) (Table 4 and Table 5). However, age-specific trends diverged (Figure 3 and Figure 4). The incidence of early-onset cases increased (AAPC 0.68, 95% CI: 0.54, 0.82), while the incidence of late-onset disease decreased (AAPC −0.47, 95% CI: −0.70, −0.23) (Table 6). The increase in early-onset breast cancer was primarily driven by rising rates among those aged 20–39 years from 2010 to 2023 (APC 1.10; 95% CI: 0.94, 1.27) and women aged 40–49 years after 2013 (APC 1.40; 95% CI: 1.13, 1.66) (Table S4).

**Figure 5:**
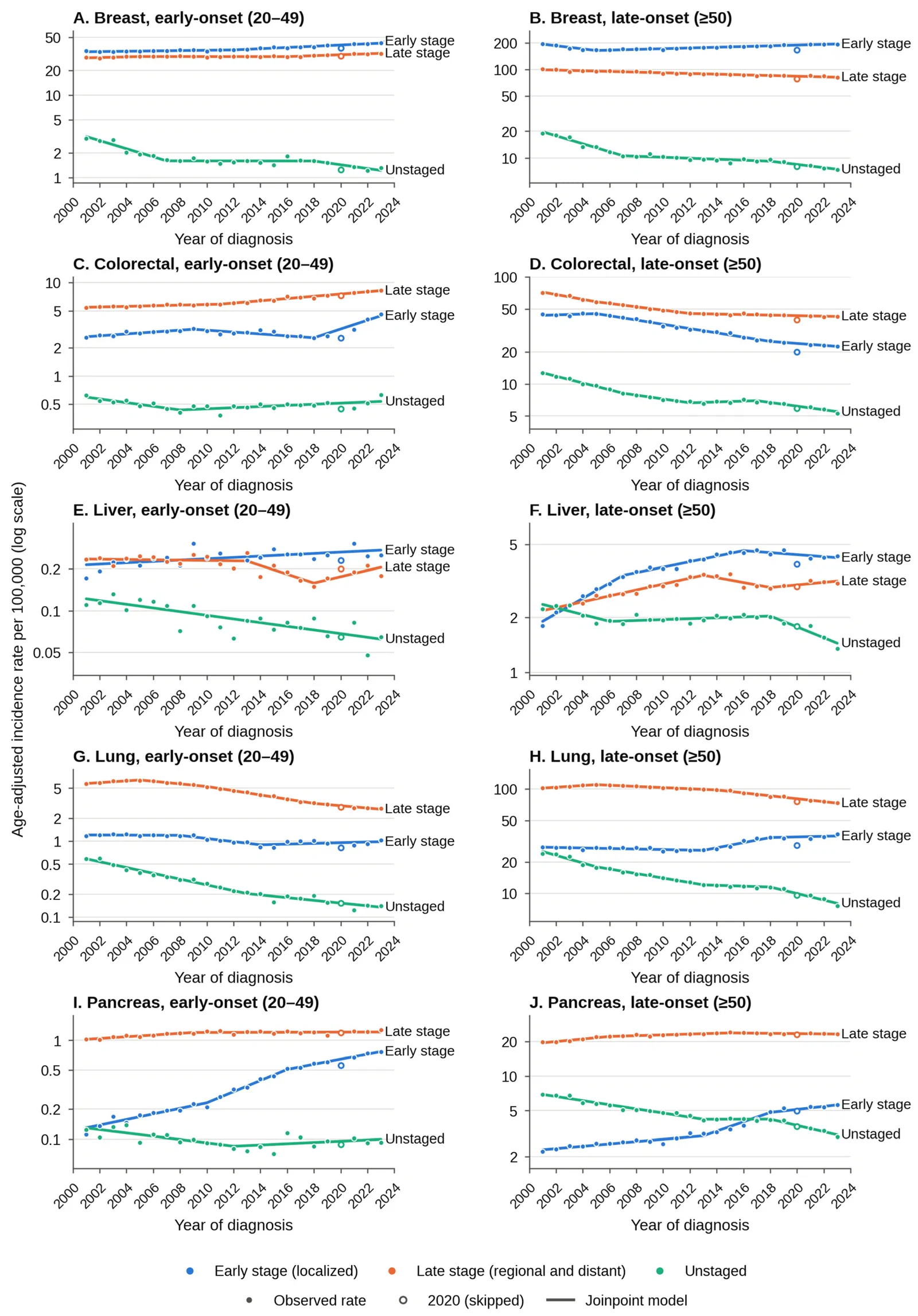
Trends in age-adjusted incidence rates of six major cancers among women in the United States from 2001 to 2023 by age of onset and stage at diagnosis. Footnotes: Due to disruptions in cancer diagnosis and reporting, 2020 data point was excluded from the trend analysis.

**Figure 6:**
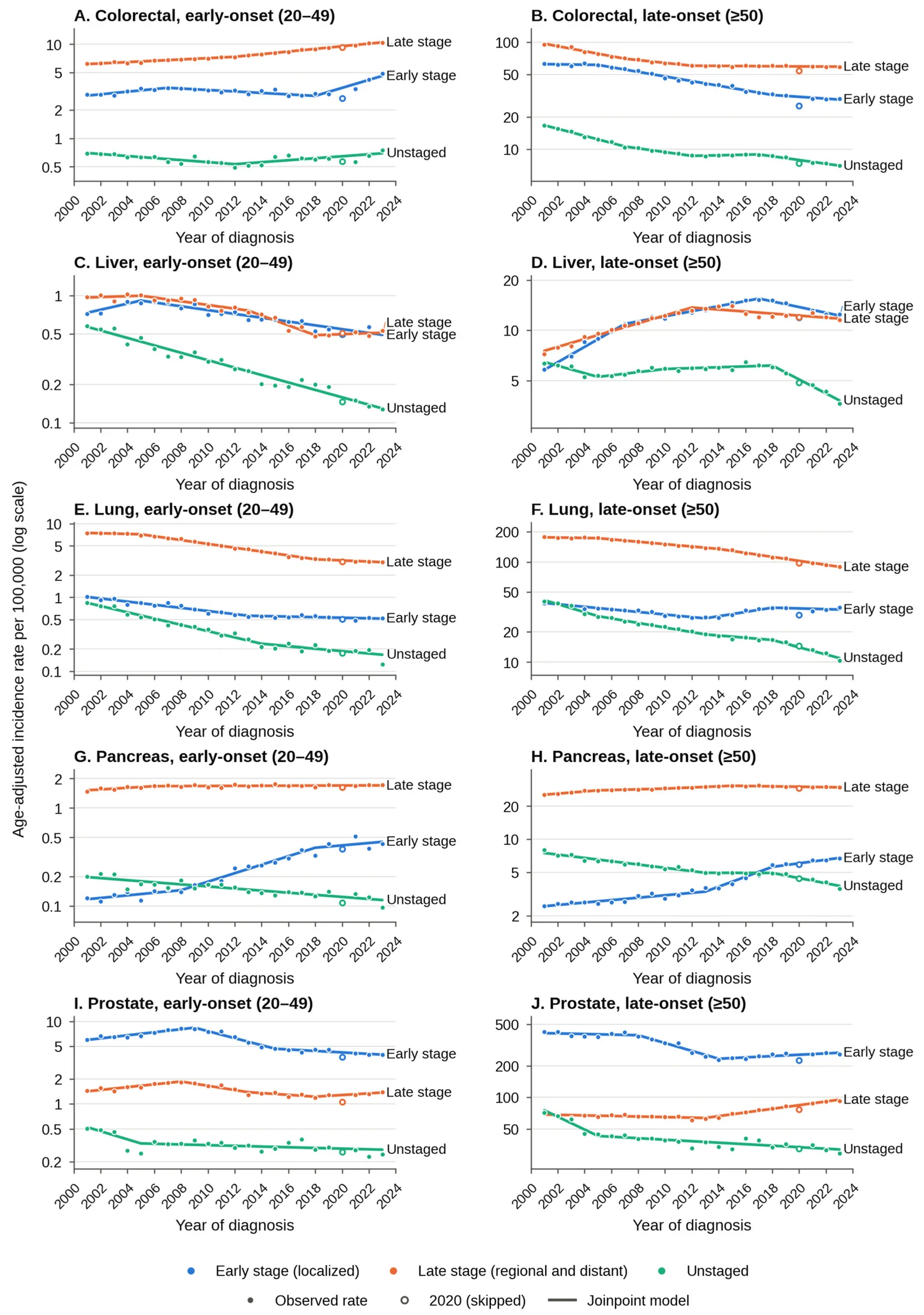
Trends in age-adjusted incidence rates of six major cancers among men in the United States from 2001 to 2023 by age of onset and stage at diagnosis. Footnotes: Due to disruptions in cancer diagnosis and reporting, 2020 data point was excluded from the trend analysis.

Stage-specific analyses revealed varying patterns by age at onset (Figure 5 and Table S6). Early- and late-stage disease incidence increased among women with early-onset breast cancer but declined in those with late-onset disease (AAPC −0.88; 95% CI: −1.00, −0.77) (Figure 6 and Table S11).

### 3.2. Colorectal Cancer Incidence and Trends

An estimated 2,643,036 individuals were diagnosed with invasive colorectal cancer during the study period (Table 2). Early-onset colorectal cancer accounted for approximately 11% of cases (Table 3, Tables S2 and S3). AAIRs were 40.72 (95% CI: 40.65–40.79) per 100,000 women and 52.63 (95% CI: 52.54–52.71) per 100,000 men (Table 2).

**Figure 7:**
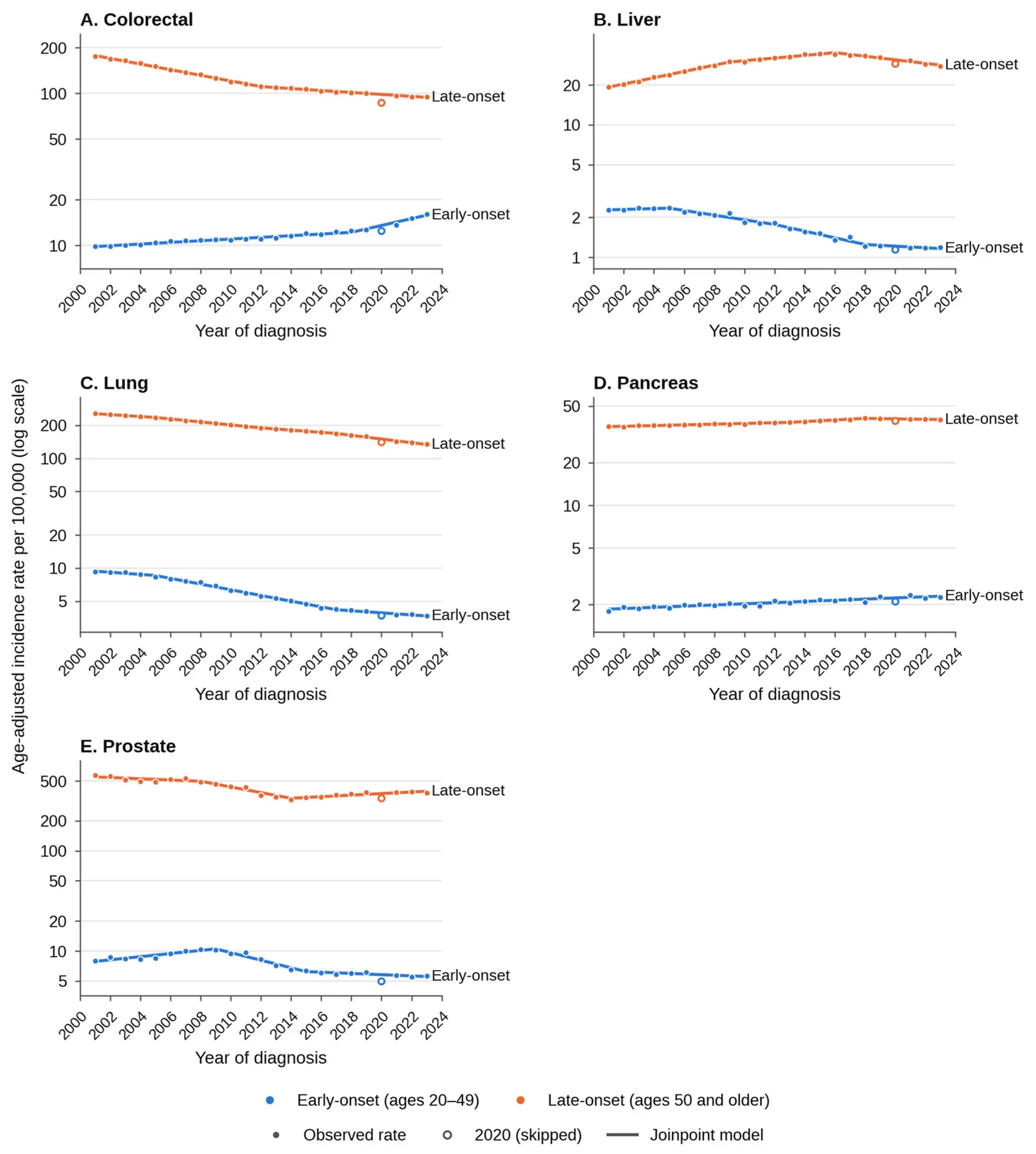
Trends in age-adjusted incidence rates of six major cancers among men aged ≥20 years in the United States from 2001 to 2023 by age of onset. Footnotes: Due to disruptions in cancer diagnosis and reporting, 2020 data point was excluded from the trend analysis.

Colorectal cancer incidence declined significantly overall (AAPC −2.02, 95% CI: −2.22, −1.81), and for both women (AAPC −2.00; 95% CI: −2.24, −1.77) and men (AAPC −2.15; 9% CI: −2.37, −1.93) (Figure 1 and Figure 2 and Table 4 and Table 5). These declines were driven by long-term reductions in late-onset colorectal cancer incidence throughout the study period (Figure 7 and Table 6).

In contrast, early-onset colorectal cancer incidence increased substantially among both women (AAPC 1.94; 95% CI: 1.57, 2.30) and men (AAPC: 2.19; 95% CI: 1.89, 2.49) (Figure 3 and Table 6, Tables S8 and S9). The most pronounced increases occurred in the most recent years of the study, specifically from 2018 to 2023, when incidence rose by 5.63% (95% CI: 4.10, 7.18) annually among women and by 5.35% (95% CI: 4.10, 6.63) annually among men (Table 6, Tables S8 and S9). Similar patterns were observed across age groups younger than 50 years (Figure 4 and Table S10).

Stage-specific data showed contrasting trends by age at onset. Late-stage colorectal cancer increased among both women (AAPC 1.89; 95% CI: 1.64, 2.14) and men (AAPC 2.45; 95% CI: 2.31, 2.60) with early-onset disease but declined in those with late-onset disease (women (AAPC −2.36; 95% CI: −2.65, −2.07) and men (AAPC −2.22; 95% CI: −2.61, −1.83)) (Figure 6 and Tables S11 and S12).

### 3.3. Liver Cancer Incidence and Trends

From 2001 to 2023, nearly 468,728 individuals were diagnosed with invasive liver cancer (Table 2). Most cases were in men (74.94%), and early-onset disease accounted for less than 10% of cases (Table 3, Tables S2 and S3). The AAIRs were 3.70 (95% CI: 3.67, 3.72) per 100,000 women and 12.58 (95% CI: 12.54, 12.62) per 100,000 men.

Overall liver cancer incidence increased (AAPC 1.33; 95% CI: 1.06, 1.59) over the study period, as did incidence among both women (AAPC 1.25; 95% CI: 0.97, 1.52) and men (AAPC 1.23; 95% CI: 0.99, 1.47) (Figure 1 and Figure 2 and Table 4 and Table 5). However, joinpoint analyses suggested that these increases occurred in the early years of the study, before declining. For example, incidence increased rapidly from 2001 to 2014 (APC 2.97; 95% CI: 2.61, 3.33) among women and from 2001 to 2009 (APC 4.64; 95% CI: 4.20, 5.08) among men (Table 5).

Age-specific incidence varied. While early-onset disease incidence declined over time in both women and men, late-onset disease increased in both women (AAPC 1.44; 95% CI: 1.19, 1.70) and men (AAPC 1.75; 95% CI: 1.46, 2.05) (Figure 3, and Table 6, Tables S8 and S9).

Stage-specific analyses largely mirrored the overall trends. Among men, late-stage early-onset liver cancer incidence decreased over time (AAPC −2.86; 95% CI: −4.52, −1.17), whereas late-stage late-onset diagnosis increased (AAPC 2.04; 95% CI: 1.50, 2.59). Among women, late-stage early-onset liver cancer incidence did not change significantly, while late-stage late-onset incidence increased (AAPC 1.70; 95% CI: 0.95, 2.46) over the study period (Figure 5 and Figure 6 and Tables S11 and S12).

### 3.4. Lung/Bronchial Cancer Incidence and Trends

Approximately 3,811,610 individuals were diagnosed with invasive lung/bronchial cancer during the study period (Table 2). Early-onset cancers accounted for nearly 5% of the cases (Table 3, Tables S2 and S3). The AAIRs were 56.82 (95% CI: 56.74, 56.91) per 100,000 women and 76.28 (95% CI: 76.17, 76.39) per 100,000 men.

Lung/bronchial cancer incidence decreased significantly overall (AAPC −2.18, 95% CI: −2.38, −1.98), and in both women (AAPC −1.38; 95% CI: −1.62, −1.15) and men (AAPC −2.97; 95% CI: −3.14, −2.79). The observed declines began early in the study and continued throughout (Figure 1 and Figure 2 and Table 4 and Table 5).

Long-term decreases occurred in both early- and late-onset disease. Among women, incidence declined for early-onset disease (AAPC −3.20; 95% CI: −3.74, −2.65) and late-onset disease (AAPC −1.26; 95% CI: −1.47, −1.06) (Figure 3 and Table 6). Similarly, incidence decreased in men with early-onset disease (AAPC −4.18, 95% CI: −4.58, −3.79) and late-onset disease (AAPC −2.90, 95% CI: −3.06, −2.74) (Table S9). Incidence declined across all age groups (Figure 4 and Table S10).

Stage-specific trends showed substantial reductions in both early- and late-stage diseases in younger adults regardless of sex and a long-term increase in early-stage incidence among women with late-onset disease (AAPC 1.13; 95% CI: 0.39, 1.88), primarily driven by a steep increase from 2013 to 2018 (APC 5.82; 95% CI: 3.01, 8.70) (Figure 5 and Figure 6 and Tables S11 and S12).

### 3.5. Pancreatic Cancer Incidence and Trends

An estimated 841,705 individuals were diagnosed with invasive pancreatic cancer from 2001 to 2023 (Table 2). Early-onset cancers accounted for approximately 6% of cases (Table 3, Tables S2 and S3). The AAIRs were 13.02 (95% CI: 12.98, 13.06) per 100,000 women and 16.26 (95% CI: 16.21, 16.31) per 100,000 men (Table 2).

Overall pancreatic cancer incidence increased slightly but significantly (AAPC 0.62; 95% CI: 0.44, 0.80), as did incidence among women (AAPC 0.66; 95% CI: 0.41, 0.91) and men (AAPC 0.55; 95% CI: 0.44, 0.66). Despite an increase in incidence throughout most of the study period, no statistically significant change was observed from 2018 to 2023 (Figure 1 and Figure 2 and Table 4 and Table 5).

Both early- and late-onset pancreatic cancer increased over time (Figure 3 and Table 6). The largest increase was among women with early-onset disease (AAPC 2.29; 95% CI: 2.15, 2.44), while men with early-onset or late-onset disease also saw smaller but significant increases (Tables S8 and S9).

Late-stage pancreatic cancer incidence increased in both women (AAPC 0.78; 95% CI: 0.43, 1.14) and men (AAPC 0.80; 95% CI: 0.52, 1.09). Nonetheless, among men, late-stage incidence began declining after 2015 (APC −0.42, 95% CI: −0.59, −0.25) (Figure 5 and Figure 6 and Tables S11 and S12).

### 3.6. Prostate Cancer Incidence and Trends

From 2001 to 2023, approximately 4,596,674 men aged ≥20 years were diagnosed with invasive prostate cancer (Table 2). Early onset accounted for < 3% of all cases (Table 3 and Table S3). The AAIR was 166.15 (95% CI: 165.99, 166.30) per 100,000 men.

Overall prostate cancer incidence declined during the study period (AAPC −1.50; 95% CI: −2.54, −0.45) (Figure 1 and Figure 2 and Table 4 and Table 5). However, trends varied over time. Incidence decreased rapidly between 2008 and 2014 (APC −6.36, 95% CI: −9.22, −3.41) before increasing thereafter, with a significant upward trend from 2014 to 2023 (APC 1.71, 95% CI: 0.40, 3.04).

Although long-term incidence declined overall, joinpoint analyses revealed an inflection point in 2014, after which late-onset incidence increased significantly (APC 1.83, 95% CI: 0.53, 3.14) (Table 6). Similar trends were observed across all age groups of individuals aged ≥50 years (Table S10), while early-onset cases declined (Figure 3 and Table S9).

Stage-specific analyses identified increasing incidence of late-stage prostate cancer among men with late-onset disease (AAPC 1.50, 95% CI: 1.17, 1.84). Specifically, there was an inflection point in 2013, followed by a substantial rise in late-stage incidence (APC 4.12, 95% CI: 3.57, 4.66) in the remaining study period, after an initial decline in late-stage cases (Figure 6 and Table S12).

## 4. Discussion

In this comprehensive population-based analysis of six major cancers in the United States from 2001 to 2023, we observed marked heterogeneity in incidence trends based on cancer type, age at onset, and stage at diagnosis. While incidence rates of lung and colorectal cancers declined substantially over the study period, particularly among older adults, the incidence of early-onset colorectal, pancreatic, and breast cancers increased, underscoring a growing cancer burden among younger adult populations. Liver cancer incidence increased during much of the study period but declined in recent years. Prostate cancer incidence revealed a unique pattern of rapid initial decline, followed by a modest rebound starting in 2014. Notably, trends of early-onset cancers diverged from those of late-onset cancers. Early-onset colorectal and breast cancers increased, whereas late-onset diseases declined. In contrast, early-onset liver, lung/bronchial, and prostate cancers decreased significantly. Across several cancers, we found notable shifts in stage at diagnosis, with reductions in the incidence of late-stage diseases. These findings highlight the uneven progress in population-level cancer prevention and emerging challenges of early-onset cancers in the U.S.

### 4.1. Breast Cancer

The overall incidence of female breast cancer did not change significantly over time, but we found stark differences by age at onset. Early-onset breast cancer increased, while late-onset disease declined modestly. The rising burden of early-onset breast cancer in this study is consistent with findings from other studies [30,31], and the underlying mechanisms remain incompletely understood. Possible explanations include increasing prevalence of known risk factors, including obesity, sedentary lifestyle, alcohol consumption, reproductive factors, hormone therapy, and environmental exposures [30,32]. Probable explanations, consistent with prior studies, for the observed increase in incidence of early-stage breast cancer in both younger and older women over time include improvements in population-based screening programs, imaging technologies, and diagnostic practices [33]. These findings underscore the need for further investigation into the drivers of early-onset breast cancer and the development of prevention strategies targeting younger women.

### 4.2. Colorectal Cancer

Colorectal cancer showed notable age-specific contrasts in this study, in alignment with other studies [34,35]. Consistent with prior studies, plausible explanations for the observed declining rates in older adults include increasing uptake of population-based screening for precancerous lesions, which, if detected, can be treated before malignant transformation [36,37,38], along with the availability of non-invasive, home-based screening options, such as multitarget stool DNA testing [39]. In contrast, the rise in early-onset colorectal cancer, particularly in recent years, is concerning. Studies have suggested that the risk factors for early-onset and late-onset colorectal cancers may differ. Although the causes of early-onset colorectal cancer remain incompletely understood, emerging evidence suggests that it may be related to obesity, alcohol consumption, metabolic dysfunction, diets, changes in the gut microbiome, environmental exposures, and physical inactivity [40]. The accompanying increase in late-stage early-onset colorectal cancer incidence highlights urgent interventions. The current guidelines from the American Cancer Society recommend that screening for colorectal cancer should begin at 45 for those with an average risk [41]. Because most early-onset cases are sporadic and affected individuals may not be eligible for screening, additional research is needed on early detection strategies for younger adults, including disease biomarkers, risk stratification, and timely clinical assessment, as modifiable risk factors continue to be investigated.

### 4.3. Liver Cancer

Liver cancer showed long-term increases in incidence followed by declines in more recent years. The finding updates the reports from prior studies using older data [42,43,44]. The earlier increase in incidence possibly reflects the long-lasting burden of chronic hepatitis B and C virus infections, alcohol-associated liver disease, and growing burden of metabolic dysfunction-associated steatotic liver disease (MASLD) [45,46]. The recent decline in liver cancer may be partly explained by the additive impact of population-level public health interventions, including hepatitis B vaccination programs, improved screening programs for viral hepatitis, safer blood transfusion practices, and effective antiviral therapies for hepatitis C virus infections [47]. Nevertheless, continued increases in incidence among older adults and increased late-stage disease incidence suggest that liver cancer remains a substantial public health burden. The growing prevalence of MASLD [48,49] may partially explain the observed pattern and could influence future trends in liver cancer incidence. However, the observational nature of the current study, which focuses on incidence trends, prevents determination of the relative contributions of these factors.

### 4.4. Lung/Bronchial Cancer

The observed sustained decline in lung cancer incidence is consistent with prior studies [50]. The observed trend may be partly explained by population-level interventions that have led to demonstrable reductions in cigarette smoking over the past decades, including tobacco control (taxation and smoke-free policies) and smoking cessation programs [51,52]. Long-term reductions in lung cancer incidence in younger adults may suggest lower smoking initiation rates at the population level according to the existing literature [53,54,55]. Based on findings from prior studies, there may be a temporal coincidence between the observed sharp increase in early-stage incidence in older women from 2013 to 2018 and the implementation of the 2013 U.S. Prevention Services Task Force (USPSTF) lung cancer screening guidelines for eligible individuals with high risk and changes in healthcare coverage policies for the screening around the same time [56,57]. However, the USCS dataset did not measure lung screening exposure. Therefore, our study cannot directly attribute the observed trend to the impact of screening interventions. Nevertheless, continued public health intervention for prevention and early detection could further reduce the huge burden of lung cancer in the U.S.

### 4.5. Pancreatic Cancer

Overall, pancreatic cancer showed long-term increases in incidence. These findings, aligning with previous studies [58], are alarming because pancreatic cancer is biologically aggressive and one of the most lethal cancers in humans [59], with no current population-wide screening program. Consistent with prior studies, possible explanations for the increase in incidence in both younger and older adults, with the greatest increase in younger women, may include rising prevalence of known risk factors, such as obesity, diabetes mellitus, and MASLD [60,61]. Although the incidence of pancreatic cancers increased over time, we found substantial increases in early-stage incidence in both women and men. Our results underscore the urgent need for targeted interventions to improve prevention through risk reduction and early detection. Recent studies suggest that, despite the aggressiveness of pancreatic cancer and its rapid progression, the disease undergoes multiple carcinogenic processes over several years, approximately a decade [62,63,64]. It is critical to prioritize research to identify opportunities for detecting pre-invasive and early-stage pancreatic tumors.

### 4.6. Prostate Cancer

The observed increase in prostate cancer incidence starting from 2014 after an initial sharp decline from 2008 to 2014, particularly in older men, may be partly explained by changing screening guidelines. The substantial decline in 2008 likely corresponds to the changing USPSTF recommendations that discouraged routine screening [65,66,67]. Late-stage incidence markedly increased from 2013, mirroring the implementation of the 2012 USPSTF guidelines that advised against annual PSA-based screening for prostate cancer in men of all ages [68]. While our observational study cannot establish causality, the temporal alignment between changing guidelines and prostate cancer trends suggests that screening practices, consistent with prior studies, may partially explain the observed trends.

### 4.7. Stage-Specific Trends Across Cancers

Across multiple cancer types, we found increases in early-stage and decreases in late-stage diagnoses. These stage-specific patterns may be explained by both improved early detection and actual changes in disease occurrence, which may vary by cancer type. Another factor that may support this pattern is stage migration, whereby more accurate staging assessments reclassify patients into different stage categories [69,70]. Therefore, changes in stage-specific trends should be interpreted cautiously, as observed trends may reflect changes in cancer incidence, screening uptake, diagnostic practices, and staging classification. Future cancer incidence trend analyses integrating screening, treatment, and mortality data may help clarify these mechanisms.

### 4.8. Implications for Policy and Clinical Practice

To address the increasing burden of these cancers, it is essential to adopt multilevel approaches that can effectively contribute to impactful cancer prevention. Thus, we recommend that policymakers prioritize (1) epidemiological, biological, and health services research to investigate modifiable risk factors for cancers and delayed detection; (2) early-life primary prevention efforts, including interventions for known modifiable lifestyle risk factors, such as obesity, diet, physical activity, alcohol consumption, and tobacco use; (3) early detection efforts, including enhancing the capacity of clinicians to recognize and treat precancerous and early-stage tumors; and (4) policies that expand healthcare access for cancer prevention, including screening and timely diagnosis.

### 4.9. Limitations and Strengths

This study has important limitations to consider when interpreting the study’s findings. First, we analyzed USCS data spanning several years. Variations in data completeness and coverage can impact trend estimates. However, the USCS ensures high-quality data through NPCR- and SEER-supported registries that resubmit and update annual data with newly gathered cancer records. Second, the USCS does not provide data on risk factors (such as smoking, alcohol consumption, and obesity), comorbidities, and healthcare access or utilization. Thus, we could not examine whether the observed trends in cancer incidence were related to temporal changes in these factors. Third, the current study was restricted to first primary malignant cancers, defined as cancers coded as one primary only or the first of two or more primaries in the USCS Sequence Number Central. As a result, subsequent primary cancers in the same individuals were excluded. Although this approach prevents individuals from contributing multiple cancers to the case counts, estimating the incidence of first primary cancers rather than all primary cancers may modestly underestimate the incidence of certain cancers among cancer survivors. Therefore, the findings of this study should be interpreted as the incidence of first primary malignant cancers rather than the incidence of all primary cancers. Fourth, we excluded the 2020 data point from joinpoint regression models based on CDC/NCI recommendations about potential bias in cancer incidence data due to COVID-19 pandemic disruptions to cancer screening, diagnosis, and reporting [22,23,24,25]. We acknowledge that incidence patterns during the immediate post-pandemic period (2021–2023) may still reflect delayed diagnoses, the recovery of screening services, and catch-up detection following the disruptions from the 2020 pandemic lockdown [24,25]. Fifth, the stage at diagnosis variable contains a category (unstaged) with unknown, missing, or unspecified stage (Table S1). This group may reflect different underlying factors, including incomplete documentation, reporting practices, or limitations in clinical information. Changes in the proportion of unstaged cases over time could influence estimates from the trend analyses. Sixth, interpreting stage-specific trends should account for potential changes in diagnostic practices, screening uptake, and stage migration over time. Sixth, national-level estimates may mask important differences across sociodemographic groups and geographic locations in the U.S. and warrant future investigation. Eighth, we could not ascertain whether patterns suggesting increasing earlier-stage diagnosis translated into reductions in cancer-specific mortality. Future studies are needed to investigate the connection. Nineth, the results of the exploratory analyses focused on narrower age categories, and stage at diagnosis should be interpreted cautiously as it is intended for hypothesis generation. Tenth, the USCS database offers only the 2000 U.S. standard population for age adjustments. A fixed standard population from 2000 may not fully reflect demographic changes between 2001 and 2023. Despite these limitations, this study analyzed data from a nationwide database, which provides high-quality data.

## 5. Conclusions

This population-based study identified heterogeneity in the patterns of cancer incidence by cancer type, age, and stage at diagnosis in the U.S. While the incidence of lung, colorectal, and more recently liver cancer declined, early-onset colorectal, pancreatic, and breast cancer continued to increase, representing an emerging challenge. These findings underscore the need for continued surveillance; research to understand risk factors; and targeted interventions, including early detection strategies, particularly for younger adults.

## Figures and Tables

**Table 1:** Annual incidence rates among individuals ≥ 20 years of age with new cancer cases in the United States, 2001 to 2023.

Cancer Type	Estimate	2001	2002	2003	2004	2005	2006	2007	2008	2009	2010	2011	2012
Breast (female)	Case Count	175,907	174,293	167,342	166,928	169,185	172,265	175,461	179,365	182,846	179,620	185,798	188,360
	Population	104,610,919	105,787,798	106,924,477	108,039,780	109,176,172	110,485,466	111,694,370	112,929,749	114,189,688	115,490,007	116,717,811	117,916,489
	AAIR (95% CI)	162.28 (161.52–163.04)	158.04 (157.30–158.79)	149.37 (148.65–150.09)	146.64 (145.93–147.35)	146.11 (145.41–146.82)	146.40 (145.71–147.10)	146.53 (145.84–147.23)	147.34 (146.65–148.03)	147.70 (147.02–148.39)	142.72 (142.04–143.39)	145.66 (144.99–146.34)	145.54 (144.87–146.21)
Colorectal	Case Count	126,569	124,646	124,532	122,436	121,080	118,933	117,495	116,345	113,434	109,994	109,309	108,474
	Population	202,073,230	204,449,149	206,665,169	209,014,464	211,338,029	213,996,734	216,405,868	218,883,056	221,385,561	224,032,119	226,671,379	229,340,838
	AAIR (95% CI)	63.43 (63.08–63.78)	61.39 (61.05–61.73)	60.22 (59.89–60.56)	58.17 (57.85–58.50)	56.42 (56.10–56.74)	54.38 (54.07–54.69)	52.56 (52.26–52.86)	50.99 (50.70–51.29)	48.69 (48.40–48.98)	46.27 (45.99–46.55)	45.16 (44.89–45.43)	43.90 (43.64–44.17)
Liver	Case Count	11,104	11,915	12,699	13,725	14,594	15,614	16,852	17,876	19,600	19,798	20,907	22,251
	Population	202,073,230	204,449,149	206,665,169	209,014,464	211,338,029	213,996,734	216,405,868	218,883,056	221,385,561	224,032,119	226,671,379	229,340,838
	AAIR (95% CI)	5.54 (5.43–5.64)	5.83 (5.72–5.93)	6.09 (5.98–6.19)	6.43 (6.32–6.54)	6.67 (6.56–6.78)	6.96 (6.85–7.07)	7.31 (7.20–7.42)	7.56 (7.45–7.67)	8.06 (7.94–8.17)	7.91 (7.79–8.02)	8.13 (8.01–8.24)	8.41 (8.30–8.52)
Lung	Case Count	162,788	163,988	166,624	165,920	168,128	167,444	166,592	167,818	167,167	164,375	163,714	164,665
	Population	202,073,230	204,449,149	206,665,169	209,014,464	211,338,029	213,996,734	216,405,868	218,883,056	221,385,561	224,032,119	226,671,379	229,340,838
	AAIR (95% CI)	81.63 (81.23–82.02)	80.80 (80.41–81.19)	80.62 (80.23–81.01)	78.95 (78.57–79.33)	78.56 (78.19–78.94)	76.82 (76.45–77.19)	74.79 (74.43–75.15)	73.59 (73.24–73.95)	71.75 (71.40–72.10)	68.98 (68.64–69.32)	67.30 (66.97–67.63)	65.95 (65.63–66.28)
Pancreas	Case Count	26,738	27,108	28,297	28,865	29,789	30,651	31,171	32,496	32,770	33,665	35,020	36,109
	Population	202,073,230	204,449,149	206,665,169	209,014,464	211,338,029	213,996,734	216,405,868	218,883,056	221,385,561	224,032,119	226,671,379	229,340,838
	AAIR (95% CI)	13.41 (13.25–13.57)	13.36 (13.20–13.52)	13.69 (13.53–13.85)	13.71 (13.55–13.87)	13.88 (13.72–14.04)	14.02 (13.86–14.18)	13.95 (13.80–14.11)	14.20 (14.04–14.36)	14.02 (13.87–14.17)	14.07 (13.92–14.22)	14.38 (14.22–14.53)	14.45 (14.30–14.60)
Prostate	Case Count	197,043	199,189	187,001	186,243	187,946	205,615	218,815	209,043	205,391	198,715	203,607	174,354
	Population	97,462,311	98,661,351	99,740,692	100,974,684	102,161,857	103,511,268	104,711,498	105,953,307	107,195,873	108,542,112	109,953,568	111,424,349
	AAIR (95% CI)	225.18 (224.18–226.19)	221.97 (220.98–222.95)	203.57 (202.64–204.51)	197.68 (196.77–198.59)	194.50 (193.61–195.39)	206.79 (205.88–207.70)	212.92 (212.01–213.83)	196.54 (195.68–197.40)	186.40 (185.58–187.23)	175.19 (174.40–175.98)	174.22 (173.45–175.00)	144.24 (143.55–144.94)
**Cancer Type**		**Estimate**	**2013**	**2014**	**2015**	**2016**	**2017**	**2018**	**2019**	**2020**	**2021**	**2022**	**2023**
Breast (female)		Case Count	193,120	196,433	201,994	204,133	207,367	211,413	218,589	199,587	226,196	226,302	228,955
		Population	119,012,905	120,148,209	121,229,417	122,288,116	123,258,580	124,131,524	124,950,717	125,709,254	126,362,506	127,436,672	128,863,335
		AAIR (95% CI)	147.10 (146.43–147.78)	147.56 (146.89–148.23)	149.57 (148.90–150.24)	148.94 (148.28–149.61)	149.38 (148.71–150.04)	150.73 (150.07–151.40)	154.10 (153.43–154.77)	139.96 (139.32–140.60)	156.47 (155.80–157.15)	154.88 (154.21–155.54)	155.08 (154.42–155.75)
Colorectal		Case Count	108,859	111,218	111,576	111,905	111,620	112,319	113,429	101,844	113,505	115,228	118,286
		Population	231,793,194	234,291,930	236,710,146	239,036,997	241,155,581	243,066,923	244,874,669	246,514,827	247,714,927	249,731,167	252,539,332
		AAIR (95% CI)	43.20 (42.94–43.46)	43.28 (43.02–43.54)	42.72 (42.47–42.98)	42.15 (41.90–42.40)	41.35 (41.11–41.60)	40.98 (40.74–41.23)	40.87 (40.62–41.11)	36.41 (36.18–36.64)	40.20 (39.96–40.44)	40.52 (40.28–40.76)	40.97 (40.73–41.21)
Liver		Case Count	23,196	24,579	25,444	25,471	25,529	25,525	25,576	23,450	25,119	24,157	23,747
		Population	231,793,194	234,291,930	236,710,146	239,036,997	241,155,581	243,066,923	244,874,669	246,514,827	247,714,927	249,731,167	252,539,332
		AAIR (95% CI)	8.57 (8.46–8.68)	8.86 (8.74–8.97)	8.96 (8.85–9.08)	8.75 (8.64–8.86)	8.65 (8.54–8.76)	8.49 (8.38–8.59)	8.37 (8.27–8.48)	7.54 (7.44–7.64)	7.99 (7.89–8.09)	7.58 (7.49–7.68)	7.34 (7.24–7.43)
Lung		Case Count	165,116	166,687	169,055	168,829	169,305	168,728	170,284	154,683	162,303	163,784	163,613
		Population	231,793,194	234,291,930	236,710,146	239,036,997	241,155,581	243,066,923	244,874,669	246,514,827	247,714,927	249,731,167	252,539,332
		AAIR (95% CI)	64.67 (64.35–64.99)	63.66 (63.35–63.97)	63.05 (62.75–63.36)	61.54 (61.24–61.84)	60.20 (59.91–60.50)	58.50 (58.21–58.78)	57.67 (57.39–57.95)	51.23 (50.97–51.49)	52.74 (52.48–53.00)	52.06 (51.80–52.32)	50.78 (50.53–51.03)
Pancreas		Case Count	36,824	38,307	39,715	40,702	41,923	43,384	44,458	43,999	45,926	46,565	47,223
		Population	231,793,194	234,291,930	236,710,146	239,036,997	241,155,581	243,066,923	244,874,669	246,514,827	247,714,927	249,731,167	252,539,332
		AAIR (95% CI)	14.43 (14.28–14.58)	14.67 (14.52–14.82)	14.94 (14.79–15.09)	14.99 (14.84–15.14)	15.12 (14.98–15.27)	15.31 (15.17–15.46)	15.41 (15.26–15.55)	14.94 (14.80–15.09)	15.40 (15.25–15.54)	15.28 (15.14–15.42)	15.17 (15.03–15.31)
Prostate		Case Count	171,946	166,585	178,223	185,481	199,980	208,403	220,421	196,014	227,435	235,074	234,150
		Population	112,780,289	114,143,721	115,480,729	116,748,881	117,897,001	118,935,399	119,923,952	120,805,573	121,352,421	122,294,495	123,675,997
		AAIR (95% CI)	138.45 (137.78–139.12)	130.33 (129.69–130.98)	135.67 (135.03–136.32)	137.70 (137.05–138.34)	144.42 (143.78–145.08)	147.39 (146.74–148.04)	152.35 (151.70–153.01)	133.06 (132.46–133.67)	151.98 (151.34–152.62)	154.30 (153.66–154.94)	150.82 (150.20–151.44)

Abbreviations: AAIR, age-adjusted incidence rate; CI, confidence interval. AAIR is per 100,000 people in the U.S. standard population for colorectal, liver, lung, and pancreatic cancer; per 100,000 women in the U.S. standard population for breast cancer; and per 100,000 men in the U.S. standard population for prostate cancer.

**Table 2:** Age-adjusted incidence rates among individuals aged ≥20 years with new cancer cases in the United States, 2001 to 2023.

Cancer Type	Case Count	AAIR (95% CI) 2001 to 2023	AAIR (95% CI) 2001	AAIR (95% CI) 2023	Absolute Change (95% CI)	Relative Change, % (95% CI)
Breast						
Female	4,431,459	149.25 (149.11–149.39)	162.28 (161.52–163.04)	155.08 (154.42–155.75)	−7.20 (−8.21 to −6.19)	−4.44 (−5.04 to −3.83)
Colorectal						
Overall	2,643,036	46.34 (46.28–46.39)	63.43 (63.08–63.78)	40.97 (40.73–41.21)	−22.46 (−22.88 to −22.04)	−35.41 (−35.93 to −34.89)
Female	1,264,358	40.72 (40.65–40.79)	55.20 (54.76–55.63)	35.65 (35.34–35.97)	−19.54 (−20.08 to −19.01)	−35.40 (−36.16 to −34.64)
Male	1,378,678	52.63 (52.54–52.71)	73.70 (73.12–74.29)	46.66 (46.29–47.03)	−27.04 (−27.73 to −26.35)	−36.69 (−37.40 to −35.98)
Liver						
Overall	468,728	7.86 (7.84–7.89)	5.54 (5.43–5.64)	7.34 (7.24–7.43)	1.80 (1.66 to 1.94)	32.52 (29.55 to 35.56)
Female	117,471	3.70 (3.67–3.72)	2.73 (2.64–2.83)	3.69 (3.59–3.78)	0.95 (0.82 to 1.09)	34.86 (29.11 to 40.86)
Male	351,257	12.58 (12.54–12.62)	8.89 (8.70–9.09)	11.46 (11.29–11.64)	2.57 (2.31 to 2.83)	28.91 (25.50 to 32.42)
Lung						
Overall	3,811,610	65.39 (65.32–65.46)	81.63 (81.23–82.02)	50.78 (50.53–51.03)	−30.85 (−31.32 to −30.38)	−37.79 (−38.22 to −37.36)
Female	1,803,323	56.82 (56.74–56.91)	64.34 (63.87–64.81)	48.01 (47.68–48.34)	−16.33 (−16.91 to −15.75)	−25.38 (−26.13 to −24.62)
Male	2,008,287	76.28 (76.17–76.39)	104.89 (104.20–105.58)	54.31 (53.93–54.69)	−50.58 (−51.37 to −49.79)	−48.22 (−48.72 to −47.72)
Pancreas						
Overall	841,705	14.55 (14.52–14.59)	13.41 (13.25–13.57)	15.17 (15.03–15.31)	1.76 (1.55 to 1.97)	13.14 (11.44 to 14.86)
Female	414,921	13.02 (12.98–13.06)	11.98 (11.78–12.18)	13.63 (13.45–13.82)	1.66 (1.39 to 1.93)	13.85 (11.44 to 16.32)
Male	426,784	16.26 (16.21–16.31)	15.05 (14.79–15.31)	16.86 (16.65–17.08)	1.81 (1.47 to 2.15)	12.06 (9.66 to 14.51)
Prostate						
Male	4,596,674	166.15 (165.99–166.30)	225.18 (224.18–226.19)	150.82 (150.20–151.44)	−74.36 (−75.55 to −73.18)	−33.02 (−33.43 to −32.62)

Abbreviations: AAIR, age-adjusted incidence rate; CI, confidence interval. AAIR is per 100,000 people in the U.S. standard population for colorectal, liver, lung, and pancreatic cancer; per 100,000 women in the U.S. standard population for breast cancer; and per 100,000 men in the U.S. standard population for prostate cancer. The absolute change was determined by calculating the numerical difference between the age-adjusted incidence rates (AAIRs) of the final year (2023) and the first year (2001). The percent relative change was calculated by dividing this absolute change by the rate from 2001 and then multiplying the result by 100.

**Table 3:** Age-adjusted incidence rates among individuals aged ≥20 years with new cancer cases in the United States from 2001 to 2023 by age of onset.

Cancer Type	Age of Onset	Case Count	AAIR (95% CI) 2001 to 2023	AAIR (95% CI) 2001	AAIR (95% CI) 2023	Absolute Change (95% CI)	Relative Change (95% CI)
Female breast	Early-Onset	950,953	67.88 (67.74–68.02)	65.86 (65.22–66.50)	75.88 (75.18–76.57)	10.01 (9.07 to 10.96)	15.20% (13.68 to 16.76)
	Late-Onset	3,480,506	277.85 (277.56–278.15)	314.68 (313.00–316.37)	280.27 (278.96–281.58)	−34.41 (−36.54 to −32.28)	−10.93% (−11.56 to −10.30)
Colorectal	Early-Onset	301,467	10.71 (10.67–10.75)	9.21 (9.04–9.38)	14.75 (14.54–14.97)	5.54 (5.26 to 5.81)	60.14% (56.41 to 63.95)
	Late-Onset	2,341,569	102.64 (102.51–102.77)	149.11 (148.25–149.98)	82.40 (81.88–82.92)	−66.71 (−67.72 to −65.71)	−44.74% (−45.21 to −44.27)
Liver	Early-Onset	32,796	1.15 (1.14–1.16)	1.38 (1.32–1.45)	0.839 (0.788–0.891)	−0.54 (−0.63 to −0.46)	−39.30% (−43.83 to −34.40)
	Late-Onset	435,932	18.48 (18.42–18.53)	12.11 (11.86–12.35)	17.61 (17.38–17.84)	5.50 (5.17 to 5.84)	45.47% (42.00 to 49.02)
Lung	Early-Onset	170,735	6.02 (5.99–6.05)	8.36 (8.20–8.52)	3.77 (3.66–3.88)	−4.59 (−4.79 to −4.40)	−54.95% (−56.50 to −53.35)
	Late-Onset	3,640,875	159.22 (159.06–159.39)	197.43 (196.44–198.42)	125.09 (124.47–125.71)	−72.34 (−73.51 to −71.17)	−36.64% (−37.09 to −36.19)
Pancreas	Early-Onset	52,250	1.85 (1.83–1.86)	1.53 (1.46–1.60)	2.19 (2.11–2.28)	0.67 (0.56 to 0.78)	43.81% (35.56 to 52.56)
	Late-Onset	789,455	34.64 (34.56–34.71)	32.18 (31.78–32.59)	35.67 (35.34–36.01)	3.49 (2.97 to 4.01)	10.84% (9.13 to 12.58)
Prostate	Early-Onset	109,334	7.64 (7.60–7.69)	7.91 (7.69–8.14)	5.59 (5.40–5.78)	−2.32 (−2.61 to −2.03)	−29.31% (−32.37 to −26.12)
	Late-Onset	4,487,340	416.68 (416.28–417.07)	568.60 (566.03–571.17)	380.36 (378.78–381.94)	−188.24 (−191.25 to −185.22)	−33.11% (−33.51 to −32.69)

Abbreviations: AAIR, age-adjusted incidence rate; CI, confidence interval. AAIR is per 100,000 people in the U.S. standard population for colorectal, liver, lung, and pancreatic cancers; per 100,000 women in the U.S. standard population for breast cancer, and per 100,000 men in the U.S. standard population for prostate cancer. The absolute change was determined by calculating the numerical difference between the age-adjusted incidence rates (AAIRs) of the final year (2023) and the first year (2001). The percent relative change was calculated by dividing this absolute change by the rate from 2001 and then multiplying the result by 100. For this study, early-onset cancer was defined as cancer diagnosed between ages 20 and 49 years, whereas late-onset cancer was defined as cancer diagnosed at 50 years of age or older.

**Table 4:** APCs and AAPCs among individuals aged ≥20 years with new cancer cases in the United States from 2001 to 2023 (2020 skipped).

Cancer Type	Annual Percent Change				Average Annual Percent Change			
	Joinpoint Segment (Year)		Estimate (95% CI)	*p*-Value	Study Duration (Year)		Estimate (95% CI)	*p*-Value
	Start	End			Start	End		
Breast (female)	2001	2005	−2.91 (−4.13 to −1.67)	<0.001	2001	2023	−0.19 (−0.43 to 0.05)	0.115
	2005	2023	0.42 (0.28 to 0.56)	<0.001				
Colorectal	2001	2006	−2.91 (−3.33 to −2.49)	<0.001	2001	2023	−2.02 (−2.22 to −1.81)	<0.001
	2006	2011	−3.94 (−4.56 to −3.32)	<0.001				
	2011	2018	−1.29 (−1.63 to −0.95)	<0.001				
	2018	2023	−0.16 (−0.66 to 0.34)	0.487				
Liver	2001	2009	4.41 (3.94 to 4.89)	<0.001	2001	2023	1.33 (1.06 to 1.59)	<0.001
	2009	2015	2.24 (1.51 to 2.98)	<0.001				
	2015	2023	−2.32 (−2.66 to −1.98)	<0.001				
Lung	2001	2006	−1.14 (−1.57 to −0.71)	<0.001	2001	2023	−2.18 (−2.38 to −1.98)	<0.001
	2006	2011	−2.74 (−3.35 to −2.12)	<0.001				
	2011	2017	−1.78 (−2.21 to −1.35)	<0.001				
	2017	2023	−2.96 (−3.28 to −2.63)	<0.001				
Pancreas	2001	2005	1.10 (0.49 to 1.71)	0.002	2001	2023	0.62 (0.44 to 0.80)	<0.001
	2005	2010	0.29 (−0.27 to 0.85)	0.279				
	2010	2018	1.04 (0.83 to 1.24)	<0.001				
	2018	2023	−0.09 (−0.46 to 0.27)	0.585				
Prostate	2001	2008	−1.29 (−3.09 to 0.55)	0.154	2001	2023	−1.50 (−2.54 to −0.45)	0.005
	2008	2014	−6.36 (−9.22 to −3.41)	<0.001				
	2014	2023	1.71 (0.40 to 3.04)	0.014				

Abbreviations: APC, annual percent change; AAPC, average annual percent change; CI, confidence interval.

**Table 5:** APCs and AAPCs among individuals aged ≥20 years with new cancer cases in the United States from 2001 to 2023 (2020 skipped) by sex.

Cancer Type	Sex	Annual Percent Change				Average Annual Percent Change			
		Joinpoint Segment (Year)		Estimate (95% CI)	*p*-Value	Study Duration (Year)		Estimate (95% CI)	*p*-Value
		Start	End			Start	End		
Breast	Female	2001	2005	−2.91 (−4.13 to −1.67)	<0.001	2001	2023	−0.19 (−0.43 to 0.05)	0.115
		2005	2023	0.42 (0.28 to 0.56)	<0.001				
Colorectal	Female	2001	2006	−2.62 (−3.09 to −2.15)	<0.001	2001	2023	−2.00 (−2.24 to −1.77)	<0.001
		2006	2011	−3.93 (−4.62 to −3.23)	<0.001				
		2011	2018	−1.44 (−1.84 to −1.04)	<0.001				
		2018	2023	−0.20 (−0.77 to 0.37)	0.456				
	Male	2001	2012	−3.61 (−3.78 to −3.44)	<0.001	2001	2023	−2.15 (−2.37 to −1.93)	<0.001
		2012	2018	−1.04 (−1.65 to −0.43)	0.003				
		2018	2023	−0.24 (−0.89 to 0.43)	0.457				
Liver	Female	2001	2014	2.97 (2.61 to 3.33)	<0.001	2001	2023	1.25 (0.97 to 1.52)	<0.001
		2014	2023	−1.19 (−1.69 to −0.68)	<0.001				
	Male	2001	2009	4.64 (4.20 to 5.08)	<0.001	2001	2023	1.23 (0.99 to 1.47)	<0.001
		2009	2016	1.72 (1.20 to 2.25)	<0.001				
		2016	2023	−3.00 (−3.38 to −2.62)	<0.001				
Lung	Female	2001	2006	0.00 (−0.52 to 0.52)	0.999	2001	2023	−1.38 (−1.62 to −1.15)	<0.001
		2006	2011	−2.12 (−2.84 to −1.40)	<0.001				
		2011	2017	−1.05 (−1.54 to −0.55)	<0.001				
		2017	2023	−2.23 (−2.60 to −1.86)	<0.001				
	Male	2001	2005	−2.01 (−2.54 to −1.47)	<0.001	2001	2023	−2.97 (−3.14 to −2.79)	<0.001
		2005	2012	−3.22 (−3.48 to −2.95)	<0.001				
		2012	2017	−2.45 (−2.99 to −1.91)	<0.001				
		2017	2023	−3.73 (−4.01 to −3.45)	<0.001				
Pancreas	Female	2001	2005	1.34 (0.52 to 2.18)	0.004	2001	2023	0.66 (0.41 to 0.91)	<0.001
		2005	2010	0.22 (−0.54 to 0.98)	0.538				
		2010	2018	0.96 (0.68 to 1.24)	<0.001				
		2018	2023	0.08 (−0.43 to 0.60)	0.728				
	Male	2001	2012	0.57 (0.47 to 0.67)	<0.001	2001	2023	0.55 (0.44 to 0.66)	<0.001
		2012	2018	1.20 (0.88 to 1.51)	<0.001				
		2018	2023	−0.27 (−0.59 to 0.06)	0.100				
Prostate	Male	2001	2008	−1.29 (−3.09 to 0.55)	0.154	2001	2023	−1.50 (−2.54 to −0.45)	0.005
		2008	2014	−6.36 (−9.22 to −3.41)	<0.001				
		2014	2023	1.71 (0.40 to 3.04)	0.014				

Abbreviations: APC, annual percent change; AAPC, average annual percent change; CI, confidence interval.

**Table 6:** APCs and AAPCs among individuals aged ≥20 years with new cancer cases in the United States from 2001 to 2023 (2020 skipped) by age of onset.

Cancer Type	Age at Onset	Annual Percent Change (95% CI)				Average Annual Percent Change (95% CI)			
		Joinpoint Segment (Year)		Estimate	*p*-Value	Study Duration (Year)		Estimate	*p*-Value
		Start	End			Start	End		
Female breast	Early-Onset	2001	2012	0.09 (−0.12 to 0.30)	0.358	2001	2023	0.68 (0.54 to 0.82)	<0.001
		2012	2023	1.28 (1.06 to 1.49)	<0.001				
	Late-Onset	2001	2005	−3.63 (−4.85 to −2.38)	<0.001	2001	2023	−0.47 (−0.70 to −0.23)	<0.001
		2005	2023	0.25 (0.12 to 0.38)	0.001				
Colorectal	Early-Onset	2001	2018	1.10 (0.86 to 1.34)	<0.001	2001	2023	2.09 (1.78 to 2.39)	<0.001
		2018	2023	5.52 (4.30 to 6.75)	<0.001				
	Late-Onset	2001	2005	−3.32 (−3.85 to −2.78)	<0.001	2001	2023	−2.70 (−2.83 to −2.56)	<0.001
		2005	2012	−4.18 (−4.47 to −3.89)	<0.001				
		2012	2023	−1.51 (−1.63 to −1.39)	<0.001				
Liver	Early-Onset	2001	2005	1.03 (−0.84 to 2.93)	0.254	2001	2023	−2.26 (−2.90 to −1.61)	<0.001
		2005	2010	−3.01 (−4.74 to −1.24)	0.003				
		2010	2018	−4.31 (−5.08 to −3.52)	<0.001				
		2018	2023	−0.78 (−2.62 to 1.09)	0.376				
	Late-Onset	2001	2008	5.48 (4.69 to 6.27)	<0.001	2001	2023	1.81 (1.47 to 2.15)	<0.001
		2008	2015	3.11 (2.37 to 3.85)	<0.001				
		2015	2023	−2.38 (−2.81 to −1.95)	<0.001				
Lung	Early-Onset	2001	2006	−1.09 (−2.18 to 0.02)	0.053	2001	2023	−3.72 (−4.15 to −3.29)	<0.001
		2006	2017	−5.53 (−5.98 to −5.08)	<0.001				
		2017	2023	−2.53 (−3.73 to −1.31)	<0.001				
	Late-Onset	2001	2006	−1.14 (−1.54 to −0.73)	<0.001	2001	2023	−2.09 (−2.27 to −1.90)	<0.001
		2006	2011	−2.56 (−3.13 to −1.98)	<0.001				
		2011	2017	−1.58 (−1.97 to −1.19)	<0.001				
		2017	2023	−2.98 (−3.28 to −2.69)	<0.001				
Pancreas	Early-Onset	2001	2023	1.55 (1.46 to 1.65)	<0.001	2001	2023	1.55 (1.46 to 1.65)	<0.001
	Late-Onset	2001	2013	0.56 (0.45 to 0.68)	<0.001	2001	2023	0.49 (0.33 to 0.66)	<0.001
		2013	2018	1.12 (0.52 to 1.73)	0.001				
		2018	2023	−0.31 (−0.74 to 0.13)	0.148				
Prostate	Early-Onset	2001	2009	3.68 (2.21 to 5.18)	<0.001	2001	2023	−1.57 (−2.65 to −0.49)	0.004
		2009	2015	−8.36 (−11.16 to −5.48)	<0.001				
		2015	2023	−1.42 (−3.22 to 0.42)	0.119				
	Late-Onset	2001	2008	−1.44 (−3.24 to 0.39)	0.112	2001	2023	−1.49 (−2.53 to −0.45)	0.005
		2008	2014	−6.33 (−9.20 to −3.37)	<0.001				
		2014	2023	1.83 (0.53 to 3.14)	0.009				

Abbreviations: APC, annual percent change; AAPC, average annual percent change; CI, confidence interval.

## Data Availability

The data used in this study are publicly available and can be obtained from the USCS website at https://www.cdc.gov/united-states-cancer-statistics/index.html (accessed on 20 August 2026).

## Supplementary Materials

The following supporting information can be downloaded at https://www.mdpi.com/article/10.3390/cancers18193149/s1. Table S1: Overall and unstaged case counts of new cancer cases per year in the United States, 2001 to 2023; Table S2: Age-adjusted incidence rates among female individuals aged ≥20 years with new cancer cases in the United States from 2001 to 2023 by age of onset; Table S3: Age-adjusted incidence rates among male individuals aged ≥20 years with new cancer cases in the United States from 2001 to 2023 by age of onset; Table S4: Age-adjusted incidence rates among individuals aged ≥20 years with new cancer cases in the United States from 2001 to 2023 by age categories; Table S5: Age-adjusted incidence rates among individuals aged ≥20 years with new cancer cases in the United States from 2001 to 2023 by stage at diagnosis; Table S6: Age-adjusted incidence rates among women aged ≥20 years with new cancer cases in the United States from 2001 to 2023 by age of onset and stage at diagnosis; Table S7: Age-adjusted incidence rates among men aged ≥20 years with new cancer cases in the United States from 2001 to 2023 by age of onset and stage at diagnosis; Table S8: APCs and AAPCs among women aged ≥20 years with new cancer cases in the United States from 2001 to 2023 (2020 skipped) by age of onset; Table S9: APCs and AAPCs among men aged ≥20 years with new cancer cases in the United States from 2001 to 2023 (2020 skipped) by age of onset; Table S10: APCs and AAPCs among individuals aged ≥20 years with new cancer cases in the United States from 2001 to 2023 (2020 skipped) by age group; Table S11: APCs and AAPCs among women aged ≥20 years with new cancer cases in the United States from 2001 to 2023 (2020 skipped) by age of onset and stage at diagnosis; Table S12: APCs and AAPCs among men aged ≥20 years with new cancer cases in the United States from 2001 to 2023 (2020 skipped) by age of onset and stage at diagnosis.

## Abbreviations

The following abbreviations are used in this manuscript:

USCS	United States Cancer Statistics
NCI	National Cancer Institute
SEER	Surveillance, Epidemiology, and End Results
AAIR	Age-adjusted incidence rate
APC	Annual percentage change
AAPC	Average annual percentage change
CI	Confidence interval
USPSTF	US Prevention Services Task Force
