# Supplementary material for: Temporal Trends in Six Major Cancers: Implications for Lifestyle-Based Cancer Prevention and Early Detection Across the Life Course
Source: Cancers (Basel). 2026 Sep 29;18(19):3149. doi: 10.3390/cancers18193149 (PMC13664070; doi:10.3390/cancers18193149)
Supplement: Supplementary file 1 [file cancers-18-03149-s001.zip › cancers-4521733-supplementary.pdf]

Title: Temporal Trends in Six Major Cancers: Implications for Life-style-Based Cancer Prevention and Early Detection Across the Life Course

Supplementary Tables

Supplementary Table S1. Overall and unstaged case counts of new cancer cases per year in the United States, 2001 to 2023

Cancer Type	Estimate	2001	2002	2003	2004	2005	2006	2007	2008	2009	2010	2011	2012
Female breast	Case Count	175,907	174,293	167,342	166,928	169,185	172,265	175,461	179,365	182,846	179,620	185,798	188,360
	Unstaged	10,399	9,996	9,806	7,625	7,692	6,951	6,388	6,431	6,980	6,608	6,500	6,263
	Unstaged, %	5.9	5.7	5.9	4.6	4.5	4.0	3.6	3.6	3.8	3.7	3.5	3.3
Colorectal	Case Count	126,569	124,646	124,532	122,436	121,080	118,933	117,495	116,345	113,434	109,994	109,309	108,474
	Unstaged	11,946	11,233	10,914	9,944	9,756	9,413	8,650	8,615	8,528	8,272	8,211	8,218
	Unstaged, %	9.4	9.0	8.8	8.1	8.1	7.9	7.4	7.4	7.5	7.5	7.5	7.6
Liver	Case Count	11,104	11,915	12,699	13,725	14,594	15,614	16,852	17,876	19,600	19,798	20,907	22,251
	Unstaged	3,540	3,598	3,664	3,200	3,282	3,331	3,396	3,684	3,887	3,900	3,938	4,059
	Unstaged, %	31.9	30.2	28.9	23.3	22.5	21.3	20.2	20.6	19.8	19.7	18.8	18.2
Lung	Case Count	162,788	163,988	166,624	165,920	168,128	167,444	166,592	167,818	167,167	164,375	163,714	164,665
	Unstaged	24,314	24,153	23,238	19,460	18,750	18,689	17,498	17,039	17,168	16,628	16,020	15,672
	Unstaged, %	14.9	14.7	13.9	11.7	11.2	11.2	10.5	10.2	10.3	10.1	9.8	9.5
Pancreas	Case Count	26,738	27,108	28,297	28,865	29,789	30,651	31,171	32,496	32,770	33,665	35,020	36,109
	Unstaged	5,940	5,644	5,825	5,143	5,155	5,215	4,899	5,067	4,959	4,861	5,046	4,892
	Unstaged, %	22.2	20.8	20.6	17.8	17.3	17.0	15.7	15.6	15.1	14.4	14.4	13.5
Prostate	Case Count	197,043	199,189	187,001	186,243	187,946	205,615	218,815	209,043	205,391	198,715	203,607	174,354
	Unstaged	22,284	21,296	20,189	14,685	14,912	14,758	15,576	14,863	15,593	15,349	15,389	13,862
	Unstaged, %	11.3	10.7	10.8	7.9	7.9	7.2	7.1	7.1	7.6	7.7	7.6	8.0

Supplementary Table S1 (continued). Overall and unstaged case counts of new cancer cases per year in the United States, 2001 to 2023

Cancer Type	Estimate	2013	2014	2015	2016	2017	2018	2019	2020	2021	2022	2023	Total 2001 to 2023
Female breast	Case Count	193,120	196,433	201,994	204,133	207,367	211,413	218,589	199,587	226,196	226,302	228,955	4,431,459
	Unstaged	6,442	6,357	5,995	6,922	6,535	6,874	6,598	5,823	6,087	5,711	5,631	160,614
	Unstaged, %	3.3	3.2	3.0	3.4	3.2	3.3	3.0	2.9	2.7	2.5	2.5	3.6
Colorectal	Case Count	108,859	111,218	111,576	111,905	111,620	112,319	113,429	101,844	113,505	115,228	118,286	2,643,036
	Unstaged	8,088	8,526	8,619	9,203	9,090	8,971	8,982	8,148	8,321	8,315	8,159	208,122
	Unstaged, %	7.4	7.7	7.7	8.2	8.1	8.0	7.9	8.0	7.3	7.2	6.9	7.9
Liver	Case Count	23,196	24,579	25,444	25,471	25,529	25,525	25,576	23,450	25,119	24,157	23,747	468,728
	Unstaged	4,175	4,346	4,283	4,805	4,679	4,656	4,345	4,004	3,979	3,599	3,159	89,509
	Unstaged, %	18.0	17.7	16.8	18.9	18.3	18.2	17.0	17.1	15.8	14.9	13.3	19.1
Lung	Case Count	165,116	166,687	169,055	168,829	169,305	168,728	170,284	154,683	162,303	163,784	163,613	3,811,610
	Unstaged	14,964	14,845	14,305	15,151	14,694	15,159	14,842	13,559	13,150	12,476	10,981	382,755
	Unstaged, %	9.1	8.9	8.5	9.0	8.7	9.0	8.7	8.8	8.1	7.6	6.7	10.0
Pancreas	Case Count	36,824	38,307	39,715	40,702	41,923	43,384	44,458	43,999	45,926	46,565	47,223	841,705
	Unstaged	4,558	4,689	4,804	4,929	4,842	5,016	5,073	4,634	4,577	4,454	4,015	114,237
	Unstaged, %	12.4	12.2	12.1	12.1	11.5	11.6	11.4	10.5	10.0	9.6	8.5	13.6
Prostate	Case Count	171,946	166,585	178,223	185,481	199,980	208,403	220,421	196,014	227,435	235,074	234,150	4,596,674
	Unstaged	16,189	14,965	14,847	19,664	19,708	17,056	18,821	17,301	19,090	17,423	16,801	390,621
	Unstaged, %	9.4	9.0	8.3	10.6	9.9	8.2	8.5	8.8	8.4	7.4	7.2	8.5

Unstaged category represents cancers with staging information indicated as unknown, missing, or not applicable. Stage at diagnosis was based on the USCS-provided Merged Summary Stage variable

Supplementary Table S2. Age-adjusted incidence rates among female individuals aged ≥20 years with new cancer cases in the United States from 2001 to 2023 by age of onset

Cancer Type	Age of Onset	Case Count	AAIR (95% CI) 2001 to 2023	AAIR (95% CI) 2001	AAIR (95% CI) 2023	Absolute Change (95% CI)	Relative Change (95% CI)
Female breast	Early-Onset	950,953	67.88 (67.74–68.02)	65.86 (65.22–66.50)	75.88 (75.18–76.57)	10.01 (9.07 to 10.96)	15.20% (13.68 to 16.76)
	Late-Onset	3,480,506	277.85 (277.56–278.15)	314.68 (313.00–316.37)	280.27 (278.96–281.58)	-34.41 (-36.54 to -32.28)	-10.93% (-11.56 to -10.30)
Colorectal	Early-Onset	138,593	9.83 (9.78–9.88)	8.59 (8.36–8.82)	13.43 (13.14–13.73)	4.84 (4.47 to 5.21)	56.37% (51.05 to 61.87)
	Late-Onset	1,125,765	89.55 (89.38–89.71)	128.86 (127.81–129.92)	70.78 (70.12–71.44)	-58.08 (-59.33 to -56.84)	-45.07% (-45.75 to -44.39)
Liver	Early-Onset	7,725	0.541 (0.529–0.553)	0.515 (0.460–0.575)	0.492 (0.438–0.551)	-0.023 (-0.102 to 0.056)	-4.46% (-18.38 to 11.84)
	Late-Onset	109,746	8.68 (8.63–8.73)	6.24 (6.01–6.48)	8.73 (8.51–8.96)	2.49 (2.17 to 2.82)	39.98% (33.77 to 46.49)
Lung	Early-Onset	84,685	5.95 (5.91–5.99)	7.46 (7.24–7.67)	3.87 (3.71–4.03)	-3.59 (-3.85 to -3.32)	-48.12% (-50.64 to -45.47)
	Late-Onset	1,718,638	137.23 (137.02–137.44)	154.24 (153.07–155.42)	117.77 (116.95–118.60)	-36.47 (-37.90 to -35.04)	-23.64% (-24.43 to -22.85)
Pancreas	Early-Onset	23,263	1.64 (1.62–1.66)	1.26 (1.17–1.35)	2.13 (2.02–2.25)	0.88 (0.73 to 1.02)	69.65% (55.25 to 85.39)
	Late-Onset	391,658	31.00 (30.90–31.10)	28.91 (28.42–29.42)	31.81 (31.38–32.24)	2.90 (2.24 to 3.56)	10.02% (7.63 to 12.46)

Abbreviations: AAIR, age-adjusted incidence rate; CI, confidence interval. AAIR is per 100,000 people in the U.S. for colorectal, liver, lung and pancreatic cancers, per 100,000 women in the U.S for breast cancer, and per 100,000 men in the U.S. for prostate cancer. The absolute change was determined by calculating the numerical difference between the AAIR of the final year (2023) and the first year (2001). The percent relative change was calculated by dividing this absolute change by the rate from 2001, then multiplying the result by 100. For this study, early-onset cancer was defined as cancer diagnosed between ages 20 and 49 years, whereas late-onset cancer was defined as cancer diagnosed at 50 years of age or older.

Supplementary Table S3. Age-adjusted incidence rates among male individuals aged ≥20 years with new cancer cases in the United States from 2001 to 2023 by age of onset

Cancer Type	Age of Onset	Case Count	AAIR (95% CI) 2001 to 2023	AAIR (95% CI) 2001	AAIR (95% CI) 2023	Absolute Change (95% CI)	Relative Change (95% CI)
Colorectal	Early-Onset	162,874	11.61 (11.55–11.66)	9.85 (9.60–10.10)	16.07 (15.75–16.39)	6.22 (5.82 to 6.63)	63.18% (58.01 to 68.52)
	Late-Onset	1,215,804	117.46 (117.25–117.67)	174.64 (173.19–176.09)	95.02 (94.21–95.84)	-79.62 (-81.28 to -77.95)	-45.59% (-46.23 to -44.94)
Liver	Early-Onset	25,071	1.76 (1.74–1.78)	2.27 (2.15–2.39)	1.18 (1.10–1.27)	-1.09 (-1.23 to -0.94)	-47.85% (-52.34 to -42.94)
	Late-Onset	326,186	29.67 (29.57–29.78)	19.36 (18.89–19.84)	27.71 (27.29–28.14)	8.35 (7.71 to 8.99)	43.13% (39.06 to 47.31)
Lung	Early-Onset	86,050	6.09 (6.05–6.13)	9.29 (9.05–9.54)	3.67 (3.52–3.82)	-5.62 (-5.91 to -5.34)	-60.54% (-62.43 to -58.56)
	Late-Onset	1,922,237	187.22 (186.95–187.49)	255.98 (254.25–257.73)	134.35 (133.40–135.32)	-121.63 (-123.61 to -119.65)	-47.51% (-48.03 to -47.00)
Pancreas	Early-Onset	28,987	2.06 (2.04–2.09)	1.80 (1.69–1.91)	2.26 (2.14–2.38)	0.46 (0.30 to 0.62)	25.42% (15.83 to 35.80)
	Late-Onset	397,797	38.69 (38.57–38.81)	35.99 (35.33–36.65)	39.94 (39.42–40.47)	3.96 (3.12 to 4.80)	11.00% (8.53 to 13.52)
Prostate	Early-Onset	109,334	7.64 (7.60–7.69)	7.91 (7.69–8.14)	5.59 (5.40–5.78)	-2.32 (-2.61 to -2.03)	-29.31% (-32.37 to -26.12)
	Late-Onset	4,487,340	416.68 (416.28–417.07)	568.60 (566.03–571.17)	380.36 (378.78–381.94)	-188.24 (-191.25 to -185.22)	-33.11% (-33.51 to -32.69)

Abbreviations: AAIR, age-adjusted incidence rate; CI, confidence interval. AAIR is per 100,000 people in the U.S. for colorectal, liver, lung and pancreatic cancers, per 100,000 women in the U.S for breast cancer, and per 100,000 men in the U.S. for prostate cancer. The absolute change was determined by calculating the numerical difference between the AAIR of the final year (2023) and the first year (2001). The percent relative change was calculated by dividing this absolute change by the rate from 2001, then multiplying the result by 100. For this study, early-onset cancer was defined as cancer diagnosed between ages 20 and 49 years, whereas late-onset cancer was defined as cancer diagnosed at 50 years of age or older.

Supplementary Table S4. Age-adjusted incidence rates among individuals aged ≥20 years with new cancer cases in the United States from 2001 to 2023 by age categories

Cancer Type	Age Category	Case Count	AAIR (95% CI) 2001 to 2023	AAIR (95% CI) 2001	AAIR (95% CI) 2023	Absolute Change (95% CI)	Relative Change, % (95% CI)
Breast (female)	20-39	235,986	26.49 (26.39–26.60)	25.65 (25.15–26.15)	29.10 (28.58–29.62)	3.45 (2.73 to 4.17)	13.45 (10.49 to 16.49)
	40-49	714,967	143.90 (143.57–144.23)	139.73 (138.17–141.31)	161.80 (160.08–163.54)	22.07 (19.74 to 24.40)	15.80 (14.02 to 17.60)
	50-59	1,040,505	217.81 (217.39–218.23)	254.61 (252.18–257.05)	227.49 (225.43–229.57)	-27.12 (-30.31 to -23.92)	-10.65 (-11.82 to -9.46)
	60-69	1,143,736	308.92 (308.35–309.49)	342.59 (339.12–346.09)	307.75 (305.37–310.14)	-34.84 (-39.06 to -30.62)	-10.17 (-11.31 to -9.01)
	70+	1,296,265	325.67 (325.11–326.24)	365.04 (362.01–368.09)	322.17 (319.81–324.54)	-42.87 (-46.72 to -39.02)	-11.74 (-12.72 to -10.76)
Colorectal	20-39	76,576	4.23 (4.20–4.26)	3.44 (3.31–3.57)	5.04 (4.89–5.19)	1.60 (1.40 to 1.80)	46.59 (39.70 to 53.82)
	40-49	224,891	22.62 (22.53–22.72)	19.82 (19.40–20.24)	32.59 (32.05–33.14)	12.77 (12.08 to 13.46)	64.45 (60.07 to 68.95)
	50-59	530,505	56.54 (56.38–56.69)	60.95 (60.10–61.81)	59.65 (58.90–60.41)	-1.29 (-2.43 to -0.16)	-2.12 (-3.95 to -0.26)
	60-69	657,033	92.96 (92.73–93.18)	139.73 (138.11–141.36)	78.26 (77.39–79.13)	-61.47 (-63.31 to -59.63)	-43.99 (-44.88 to -43.09)
	70+	1,154,011	165.95 (165.65–166.25)	262.95 (260.96–264.95)	113.13 (112.08–114.19)	-149.82 (-152.07 to -147.56)	-56.98 (-57.49 to -56.46)
Liver	20-39	6,933	0.37 (0.37–0.38)	0.37 (0.33–0.42)	0.38 (0.34–0.42)	0.00 (-0.06 to 0.06)	0.71 (-13.99 to 17.91)
	40-49	25,863	2.57 (2.54–2.60)	3.23 (3.07–3.41)	1.69 (1.57–1.82)	-1.54 (-1.75 to -1.33)	-47.78 (-52.30 to -42.84)
	50-59	119,751	12.51 (12.44–12.59)	7.60 (7.30–7.90)	7.32 (7.07–7.58)	-0.28 (-0.67 to 0.12)	-3.64 (-8.61 to 1.60)
	60-69	162,213	22.86 (22.75–22.97)	12.93 (12.44–13.43)	22.86 (22.40–23.34)	9.94 (9.26 to 10.62)	76.89 (69.40 to 84.71)
	70+	153,968	22.19 (22.08–22.30)	16.90 (16.39–17.41)	25.86 (25.36–26.36)	8.96 (8.25 to 9.67)	53.06 (47.71 to 58.60)
Lung	20-39	24,016	1.33 (1.32–1.35)	1.85 (1.76–1.95)	1.24 (1.16–1.31)	-0.62 (-0.74 to -0.49)	-33.23 (-38.32 to -27.71)
	40-49	146,719	14.63 (14.55–14.70)	20.31 (19.89–20.74)	8.41 (8.14–8.69)	-11.90 (-12.41 to -11.40)	-58.59 (-60.18 to -56.94)
	50-59	592,769	61.83 (61.67–61.98)	82.16 (81.17–83.16)	41.69 (41.08–42.31)	-40.47 (-41.63 to -39.30)	-49.25 (-50.21 to -48.28)
	60-69	1,172,879	166.26 (165.96–166.57)	224.88 (222.83–226.95)	140.75 (139.59–141.91)	-84.13 (-86.49 to -81.77)	-37.41 (-38.18 to -36.64)
	70+	1,875,227	271.17 (270.79–271.56)	314.76 (312.58–316.94)	213.30 (211.86–214.75)	-101.45 (-104.07 to -98.84)	-32.23 (-32.89 to -31.57)
Pancreas	20-39	11,343	0.62 (0.61–0.64)	0.41 (0.36–0.45)	0.87 (0.81–0.93)	0.46 (0.38 to 0.54)	113.34 (87.15 to 143.19)
	40-49	40,907	4.10 (4.06–4.14)	3.58 (3.41–3.76)	4.63 (4.43–4.84)	1.05 (0.78 to 1.32)	29.31 (20.95 to 38.25)
	50-59	133,277	13.95 (13.88–14.03)	12.54 (12.16–12.93)	14.63 (14.26–15.00)	2.09 (1.55 to 2.62)	16.65 (12.10 to 21.38)
	60-69	232,268	32.89 (32.76–33.02)	29.89 (29.15–30.65)	35.10 (34.52–35.68)	5.21 (4.26 to 6.15)	17.42 (13.95 to 21.00)
	70+	423,910	60.98 (60.80–61.17)	57.71 (56.78–58.65)	61.53 (60.75–62.31)	3.82 (2.61 to 5.03)	6.62 (4.45 to 8.83)
Prostate	20-39	2,216	0.26 (0.25–0.27)	0.25 (0.20–0.30)	0.15 (0.12–0.20)	-0.10 (-0.16 to -0.03)	-38.30 (-55.09 to -15.24)
	40-49	107,118	21.21 (21.08–21.34)	21.98 (21.36–22.62)	15.58 (15.05–16.12)	-6.40 (-7.23 to -5.58)	-29.13 (-32.22 to -25.89)
	50-59	884,375	187.09 (186.69–187.48)	223.53 (221.19–225.89)	159.96 (158.26–161.68)	-63.57 (-66.47 to -60.66)	-28.44 (-29.50 to -27.36)
	60-69	1,851,514	550.48 (549.69–551.28)	711.62 (706.28–716.98)	509.68 (506.50–512.87)	-201.94 (-208.16 to -195.72)	-28.38 (-29.07 to -27.67)
	70+	1,751,451	587.63 (586.75–588.50)	871.60 (865.83–877.39)	543.77 (540.34–547.22)	-327.82 (-334.54 to -321.11)	-37.61 (-38.18 to -37.04)

Abbreviations: AAIR, age-adjusted incidence rate; CI, confidence interval. AAIR is per 100,000 people in the U.S. for colorectal, liver, lung and pancreatic cancers, per 100,000 women in the U.S for breast cancer, and per 100,000 men in the U.S. for prostate cancer. The absolute change was determined by calculating the numerical difference between the AAIR of the final year (2023) and the first year (2001). The percent relative change was calculated by dividing this absolute change by the rate from 2001, then multiplying the result by 100. For this study, early-onset cancer was defined as cancer diagnosed between ages 20 and 49 years, whereas late-onset cancer was defined as cancer diagnosed at 50 years of age or older.

Supplementary Table S5. Age-adjusted incidence rates among individuals aged ≥20 years with new cancer cases in the United States from 2001 to 2023 by stage at diagnosis

Cancer Type	Stage at Diagnosis	Case Count	AAIR (95% CI) 2001 to 2023	AAIR (95% CI) 2001	AAIR (95% CI) 2023	Absolute Change (95% CI)	Relative Change, % (95% CI)
Breast (female)	Early	2,743,730	91.45 (91.34–91.56)	96.20 (95.62–96.79)	100.68 (100.15–101.21)	4.48 (3.69 to 5.27)	4.66 (3.82 to 5.50)
	Late	1,527,115	52.62 (52.53–52.70)	56.88 (56.43–57.34)	50.76 (50.37–51.15)	-6.12 (-6.72 to -5.52)	-10.76 (-11.74 to -9.77)
	Unstaged	160,614	5.18 (5.15–5.20)	9.20 (9.02–9.38)	3.64 (3.54–3.74)	-5.56 (-5.77 to -5.36)	-60.45 (-61.75 to -59.12)
Colorectal	Early	932,024	16.27 (16.24–16.30)	22.10 (21.89–22.31)	12.94 (12.81–13.08)	-9.16 (-9.40 to -8.91)	-41.43 (-42.24 to -40.60)
	Late	1,502,890	26.40 (26.36–26.44)	35.32 (35.06–35.58)	25.22 (25.03–25.41)	-10.10 (-10.42 to -9.78)	-28.60 (-29.34 to -27.84)
	Unstaged	208,122	3.66 (3.65–3.68)	6.00 (5.89–6.11)	2.80 (2.74–2.86)	-3.20 (-3.33 to -3.08)	-53.36 (-54.67 to -52.01)
Liver	Early	197,627	3.30 (3.28–3.31)	1.67 (1.62–1.73)	3.39 (3.33–3.46)	1.72 (1.64 to 1.81)	102.90 (95.17 to 110.93)
	Late	181,592	3.04 (3.02–3.05)	2.09 (2.03–2.16)	2.95 (2.89–3.01)	0.86 (0.77 to 0.94)	40.81 (35.77 to 46.05)
	Unstaged	89,509	1.53 (1.52–1.54)	1.77 (1.71–1.83)	0.990 (0.96–1.03)	-0.78 (-0.84 to -0.71)	-43.82 (-46.48 to -41.04)
Lung	Early	720,029	12.39 (12.36–12.42)	13.15 (12.99–13.31)	14.32 (14.19–14.46)	1.17 (0.96 to 1.38)	8.90 (7.25 to 10.57)
	Late	2,708,826	46.29 (46.23–46.34)	56.25 (55.92–56.58)	32.95 (32.75–33.15)	-23.30 (-23.68 to -22.91)	-41.42 (-41.91 to -40.92)
	Unstaged	382,755	6.71 (6.69–6.73)	12.23 (12.08–12.38)	3.51 (3.44–3.57)	-8.72 (-8.89 to -8.56)	-71.33 (-71.97 to -70.67)
Pancreas	Early	97,182	1.70 (1.69–1.72)	0.97 (0.93–1.02)	2.73 (2.67–2.79)	1.76 (1.69 to 1.84)	181.23 (167.60 to 195.55)
	Late	630,286	10.84 (10.81–10.86)	9.45 (9.31–9.58)	11.12 (11.00–11.24)	1.67 (1.49 to 1.85)	17.67 (15.59 to 19.79)
	Unstaged	114,237	2.01 (2.00–2.03)	2.99 (2.91–3.07)	1.32 (1.28–1.36)	-1.67 (-1.76 to -1.58)	-55.85 (-57.59 to -54.03)
Prostate	Early	3,395,205	121.36 (121.23–121.49)	168.85 (167.99–169.71)	102.68 (102.17–103.19)	-66.17 (-67.17 to -65.17)	-39.19 (-39.62 to -38.75)
	Late	810,848	29.19 (29.12–29.25)	28.45 (28.09–28.80)	36.75 (36.44–37.06)	8.31 (7.83 to 8.78)	29.20 (27.26 to 31.16)
	Unstaged	390,621	15.60 (15.55–15.65)	27.89 (27.52–28.26)	11.38 (11.21–11.56)	-16.50 (-16.92 to -16.09)	-59.18 (-60.00 to -58.34)

Abbreviations: AAIR, age-adjusted incidence rate; CI, confidence interval. AAIR is per 100,000 people in the U.S. for colorectal, liver, lung and pancreatic cancers, per 100,000 women in the U.S for breast cancer, and per 100,000 men in the U.S. for prostate cancer. The absolute change was determined by calculating the numerical difference between the AAIR of the final year (2023) and the first year (2001). The percent relative change was calculated by dividing this absolute change by the rate from 2001, then multiplying the result by 100. For this study, early-onset cancer was defined as cancer diagnosed between ages 20 and 49 years, whereas late-onset cancer was defined as cancer diagnosed at 50 years of age or older.

Supplementary Table S6. Age-adjusted incidence rates among women aged ≥20 years with new cancer cases in the United States from 2001 to 2023 by age of onset and stage at diagnosis

Cancer Type	Age of Onset	Stage at Diagnosis	Case Count	AAIR (95% CI) 2001 to 2023	AAIR (95% CI) 2001	AAIR (95% CI) 2023	Absolute Change (95% CI)	Relative Change, % (95% CI)
Breast (female)	Early-Onset	Early	512,341	36.50 (36.40–36.60)	34.18 (33.72–34.65)	42.83 (42.31–43.35)	8.64 (7.95 to 9.34)	25.28 (23.02 to 27.59)
		Late	414,058	29.62 (29.53–29.71)	28.69 (28.27–29.11)	31.74 (31.29–32.19)	3.05 (2.43 to 3.67)	10.63 (8.39 to 12.91)
		Unstaged	24,554	1.75 (1.73–1.77)	2.99 (2.85–3.13)	1.31 (1.22–1.41)	-1.68 (-1.84 to -1.51)	-56.15 (-59.65 to -52.34)
	Late-Onset	Early	2,231,389	178.30 (178.07–178.54)	194.22 (192.90–195.54)	192.11 (191.04–193.20)	-2.10 (-3.81 to -0.40)	-1.08 (-1.95 to -0.21)
		Late	1,113,057	88.96 (88.80–89.13)	101.44 (100.49–102.41)	80.83 (80.13–81.55)	-20.61 (-21.80 to -19.42)	-20.32 (-21.34 to -19.28)
		Unstaged	136,060	10.59 (10.53–10.64)	19.02 (18.62–19.43)	7.32 (7.11–7.53)	-11.70 (-12.16 to -11.25)	-61.52 (-62.87 to -60.13)
Colorectal	Early-Onset	Early	42,123	2.98 (2.95–3.01)	2.58 (2.46–2.71)	4.59 (4.42–4.76)	2.01 (1.79 to 2.22)	77.61 (67.01 to 88.89)
		Late	89,547	6.36 (6.32–6.40)	5.38 (5.20–5.57)	8.21 (7.99–8.45)	2.83 (2.54 to 3.12)	52.62 (46.07 to 59.48)
		Unstaged	6,923	0.49 (0.48–0.50)	0.62 (0.56–0.69)	0.63 (0.57–0.69)	0.00 (-0.09 to 0.09)	0.62 (-12.67 to 15.94)
	Late-Onset	Early	404,675	32.42 (32.32–32.52)	44.71 (44.09–45.34)	22.56 (22.19–22.94)	-22.15 (-22.88 to -21.43)	-49.54 (-50.62 to -48.44)
		Late	622,248	49.55 (49.43–49.68)	71.51 (70.72–72.30)	42.91 (42.40–43.42)	-28.60 (-29.53 to -27.66)	-39.99 (-40.96 to -39.01)
		Unstaged	98,842	7.58 (7.53–7.63)	12.64 (12.32–12.97)	5.30 (5.13–5.49)	-7.34 (-7.70 to -6.97)	-58.03 (-59.77 to -56.23)
Liver	Early-Onset	Early	3,429	0.24 (0.23–0.25)	0.17 (0.14–0.21)	0.25 (0.21–0.29)	0.08 (0.03 to 0.13)	46.28 (14.19 to 87.39)
		Late	3,032	0.21 (0.21–0.22)	0.23 (0.20–0.28)	0.18 (0.15–0.21)	-0.06 (-0.11 to -0.01)	-24.14 (-40.86 to -2.71)
		Unstaged	1,264	0.09 (0.08–0.09)	0.11 (0.09–0.14)	0.07 (0.05–0.09)	-0.05 (-0.08 to -0.01)	-41.33 (-60.33 to -13.22)
	Late-Onset	Early	48,009	3.81 (3.77–3.84)	1.81 (1.68–1.94)	4.31 (4.16–4.47)	2.51 (2.30 to 2.71)	138.73 (120.56 to 158.40)
		Late	37,058	2.94 (2.91–2.97)	2.21 (2.07–2.36)	3.07 (2.94–3.20)	0.86 (0.66 to 1.05)	38.79 (28.55 to 49.85)
		Unstaged	24,679	1.93 (1.91–1.96)	2.22 (2.09–2.36)	1.35 (1.27–1.44)	-0.87 (-1.03 to -0.71)	-39.09 (-44.33 to -33.36)
Lung	Early-Onset	Early	15,018	1.05 (1.04–1.07)	1.17 (1.09–1.26)	1.03 (0.95–1.11)	-0.14 (-0.26 to -0.02)	-12.15 (-21.06 to -2.23)

Cancer Type	Age of Onset	Stage at Diagnosis	Case Count	AAIR (95% CI) 2001 to 2023	AAIR (95% CI) 2001	AAIR (95% CI) 2023	Absolute Change (95% CI)	Relative Change, % (95% CI)
		Late	65,663	4.62 (4.58–4.65)	5.70 (5.51–5.89)	2.70 (2.57–2.83)	-3.00 (-3.23 to -2.77)	-52.66 (-55.36 to -49.79)
		Unstaged	4,004	0.28 (0.27–0.29)	0.59 (0.53–0.65)	0.14 (0.11–0.18)	-0.45 (-0.51 to -0.38)	-75.85 (-80.90 to -69.48)
	Late-Onset	Early	366,704	29.43 (29.33–29.53)	27.64 (27.14–28.14)	36.77 (36.32–37.24)	9.14 (8.46 to 9.81)	33.07 (30.19 to 36.01)
		Late	1,177,714	94.08 (93.91–94.26)	102.61 (101.66–103.58)	73.42 (72.77–74.07)	-29.19 (-30.35 to -28.04)	-28.45 (-29.36 to -27.52)
		Unstaged	174,220	13.72 (13.65–13.78)	23.99 (23.54–24.45)	7.58 (7.37–7.79)	-16.42 (-16.91 to -15.92)	-68.42 (-69.45 to -67.34)
Pancreas	Early-Onset	Early	5,227	0.37 (0.36–0.38)	0.11 (0.09–0.14)	0.76 (0.70–0.83)	0.65 (0.58 to 0.72)	583.07 (430.67 to 779.23)
		Late	16,629	1.17 (1.16–1.19)	1.02 (0.94–1.11)	1.28 (1.19–1.37)	0.26 (0.14 to 0.38)	25.24 (12.74 to 39.13)
		Unstaged	1,407	0.10 (0.09–0.10)	0.12 (0.10–0.16)	0.09 (0.07–0.12)	-0.03 (-0.07 to 0.01)	-25.75 (-47.23 to 4.49)
	Late-Onset	Early	46,162	3.65 (3.62–3.68)	2.22 (2.08–2.36)	5.62 (5.44–5.80)	3.40 (3.17 to 3.63)	153.39 (136.25 to 171.78)
		Late	284,633	22.68 (22.60–22.77)	19.78 (19.37–20.20)	23.21 (22.85–23.58)	3.43 (2.88 to 3.99)	17.36 (14.31 to 20.50)
		Unstaged	60,863	4.67 (4.63–4.70)	6.92 (6.68–7.16)	2.98 (2.85–3.11)	-3.94 (-4.21 to -3.67)	-56.96 (-59.30 to -54.49)

Abbreviations: AAIR, age-adjusted incidence rate; CI, confidence interval. AAIR is per 100,000 people in the U.S. for colorectal, liver, lung and pancreatic cancers, per 100,000 women in the U.S for breast cancer, and per 100,000 men in the U.S. for prostate cancer. The absolute change was determined by calculating the numerical difference between the AAIR of the final year (2023) and the first year (2001). The percent relative change was calculated by dividing this absolute change by the rate from 2001, then multiplying the result by 100. For this study, early-onset cancer was defined as cancer diagnosed between ages 20 and 49 years, whereas late-onset cancer was defined as cancer diagnosed at 50 years of age or older.

Supplementary Table S7. Age-adjusted incidence rates among men aged ≥20 years with new cancer cases in the United States from 2001 to 2023 by age of onset and stage at diagnosis

Cancer Type	Early-Onset vs. Late-Onset	Stage at Diagnosis	Case Count	AAIR (95% CI) 2001 to 2023	AAIR (95% CI) 2001	AAIR (95% CI) 2023	Absolute Change (95% CI)	Relative Change, % (95% CI)
Colorectal	Early-Onset	Early	45,359	3.23 (3.20–3.26)	2.92 (2.79–3.06)	4.88 (4.70–5.05)	1.95 (1.73 to 2.18)	66.91 (57.36 to 77.04)
		Late	108,947	7.77 (7.72–7.82)	6.23 (6.03–6.43)	10.44 (10.18–10.70)	4.21 (3.88 to 4.53)	67.59 (60.97 to 74.47)
		Unstaged	8,568	0.61 (0.60–0.62)	0.70 (0.63–0.77)	0.75 (0.68–0.82)	0.06 (-0.04 to 0.15)	8.06 (-5.33 to 23.33)
	Late-Onset	Early	439,867	42.43 (42.31–42.56)	62.91 (62.04–63.78)	29.60 (29.14–30.05)	-33.31 (-34.29 to -32.33)	-52.95 (-53.91 to -51.97)
		Late	682,148	65.33 (65.17–65.49)	95.00 (93.94–96.07)	58.37 (57.74–59.01)	-36.63 (-37.87 to -35.39)	-38.56 (-39.51 to -37.59)
		Unstaged	93,789	9.69 (9.63–9.76)	16.72 (16.26–17.20)	7.05 (6.82–7.28)	-9.67 (-10.19 to -9.16)	-57.85 (-59.61 to -56.01)
Liver	Early-Onset	Early	10,033	0.71 (0.69–0.72)	0.72 (0.65–0.79)	0.52 (0.47–0.59)	-0.19 (-0.28 to -0.11)	-27.04 (-36.83 to -15.72)
		Late	10,773	0.76 (0.74–0.77)	0.97 (0.90–1.06)	0.53 (0.48–0.59)	-0.44 (-0.54 to -0.34)	-45.37 (-52.28 to -37.47)
		Unstaged	4,265	0.30 (0.29–0.31)	0.58 (0.52–0.64)	0.13 (0.10–0.16)	-0.45 (-0.52 to -0.38)	-77.97 (-82.79 to -71.80)
	Late-Onset	Early	136,156	12.28 (12.21–12.34)	5.84 (5.59–6.10)	12.51 (12.23–12.80)	6.67 (6.29 to 7.06)	114.22 (103.87 to 125.11)
		Late	130,729	11.79 (11.73–11.86)	7.20 (6.92–7.49)	11.57 (11.30–11.85)	4.37 (3.97 to 4.76)	60.64 (53.40 to 68.22)
		Unstaged	59,301	5.60 (5.56–5.65)	6.32 (6.05–6.60)	3.63 (3.48–3.79)	-2.69 (-3.00 to -2.37)	-42.54 (-45.95 to -38.90)
Lung	Early-Onset	Early	9,507	0.67 (0.66–0.68)	1.02 (0.94–1.10)	0.52 (0.47–0.58)	-0.50 (-0.59 to -0.40)	-48.84 (-55.30 to -41.46)
		Late	71,301	5.05 (5.02–5.09)	7.43 (7.21–7.65)	3.02 (2.88–3.16)	-4.41 (-4.66 to -4.15)	-59.32 (-61.47 to -57.05)
		Unstaged	5,242	0.37 (0.36–0.38)	0.85 (0.77–0.92)	0.12 (0.10–0.16)	-0.72 (-0.80 to -0.64)	-85.32 (-88.46 to -81.34)
	Late-Onset	Early	328,800	32.43 (32.32–32.55)	38.69 (38.02–39.37)	34.84 (34.35–35.33)	-3.86 (-4.69 to -3.02)	-9.96 (-11.95 to -7.93)
		Late	1,394,148	133.89 (133.67–134.12)	177.21 (175.78–178.65)	89.12 (88.35–89.90)	-88.09 (-89.72 to -86.46)	-49.71 (-50.30 to -49.11)
		Unstaged	199,289	20.89 (20.79–20.98)	40.08 (39.37–40.80)	10.39 (10.12–10.67)	-29.69 (-30.45 to -28.92)	-74.07 (-74.89 to -73.23)
Pancreas	Early-Onset	Early	3,499	0.25 (0.24–0.26)	0.12 (0.09–0.15)	0.43 (0.38–0.49)	0.31 (0.25 to 0.37)	258.57 (176.70 to 364.66)
		Late	23,361	1.66 (1.64–1.68)	1.48 (1.39–1.58)	1.73 (1.63–1.84)	0.25 (0.11 to 0.39)	16.97 (7.00 to 27.88)
		Unstaged	2,127	0.15 (0.15–0.16)	0.20 (0.17–0.24)	0.10 (0.07–0.12)	-0.10 (-0.15 to -0.06)	-52.00 (-64.91 to -34.34)
	Late-Onset	Early	42,294	4.21 (4.17–4.25)	2.47 (2.29–2.65)	6.68 (6.47–6.90)	4.22 (3.94 to 4.49)	170.95 (150.65 to 192.90)
		Late	305,663	29.18 (29.08–29.29)	25.52 (24.98–26.07)	29.74 (29.29–30.20)	4.22 (3.52 to 4.93)	16.55 (13.55 to 19.64)
		Unstaged	49,840	5.30 (5.25–5.35)	8.00 (7.68–8.33)	3.52 (3.36–3.69)	-4.48 (-4.84 to -4.12)	-56.00 (-58.61 to -53.22)
Prostate	Early-Onset	Early	83,596	5.84 (5.81–5.88)	5.96 (5.77–6.16)	3.95 (3.79–4.11)	-2.01 (-2.26 to -1.76)	-33.75 (-37.10 to -30.23)
		Late	21,082	1.47 (1.45–1.49)	1.44 (1.35–1.54)	1.40 (1.30–1.49)	-0.05 (-0.18 to 0.09)	-3.19 (-11.95 to 6.44)
		Unstaged	4,656	0.33 (0.32–0.34)	0.51 (0.45–0.56)	0.25 (0.21–0.29)	-0.26 (-0.33 to -0.19)	-51.49 (-60.16 to -40.92)
	Late-Onset	Early	3,311,609	303.94 (303.61–304.27)	426.29 (424.09–428.50)	258.73 (257.44–260.03)	-167.56 (-170.12 to -165.01)	-39.31 (-39.74 to -38.87)

Cancer Type	Early-Onset vs. Late-Onset	Stage at Diagnosis	Case Count	AAIR (95% CI) 2001 to 2023	AAIR (95% CI) 2001	AAIR (95% CI) 2023	Absolute Change (95% CI)	Relative Change, % (95% CI)
		Late	789,766	72.99 (72.83–73.16)	71.13 (70.23–72.04)	92.64 (91.85–93.43)	21.51 (20.31 to 22.70)	30.23 (28.26 to 32.24)
		Unstaged	385,965	39.74 (39.61–39.87)	71.17 (70.22–72.13)	28.99 (28.54–29.44)	-42.18 (-43.24 to -41.12)	-59.26 (-60.09 to -58.42)

Abbreviations: AAIR, age-adjusted incidence rate; CI, confidence interval. AAIR is per 100,000 people in the U.S. for colorectal, liver, lung and pancreatic cancers, per 100,000 women in the U.S for breast cancer, and per 100,000 men in the U.S. for prostate cancer. The absolute change was determined by calculating the numerical difference between the AAIR of the final year (2023) and the first year (2001). The percent relative change was calculated by dividing this absolute change by the rate from 2001, then multiplying the result by 100. For this study, early-onset cancer was defined as cancer diagnosed between ages 20 and 49 years, whereas late-onset cancer was defined as cancer diagnosed at 50 years of age or older.

Supplementary Table S8. APCs and AAPCs among women aged ≥20 years with new cancer cases in the United States from 2001 to 2023 (2020 skipped) by age of onset

Cancer Type	Early-Onset vs. Late-Onset	Joinpoint Segment (Year)		Annual Percent Change (95% CI)	P-value	AAPC Segment (Year)		Average Annual Percent Change (95% CI)	P-value
		Start	End			Start	End		
Female breast	Early-Onset	2001	2012	0.09 (-0.12 to 0.30)	0.358	2001	2023	0.68 (0.54 to 0.82)	<0.001
		2012	2023	1.28 (1.06 to 1.49)	<0.001				
	Late-Onset	2001	2005	-3.63 (-4.85 to -2.38)	<0.001	2001	2023	-0.47 (-0.70 to -0.23)	<0.001
		2005	2023	0.25 (0.12 to 0.38)	0.001				
Colorectal	Early-Onset	2001	2018	0.87 (0.62 to 1.13)	<0.001	2001	2023	1.94 (1.57 to 2.30)	<0.001
		2018	2023	5.63 (4.10 to 7.18)	<0.001				
	Late-Onset	2001	2006	-3.03 (-3.35 to -2.70)	<0.001	2001	2023	-2.73 (-2.86 to -2.59)	<0.001
		2006	2011	-4.65 (-5.14 to -4.15)	<0.001				
		2011	2023	-1.79 (-1.88 to -1.70)	<0.001				
Liver	Early-Onset	2001	2023	-0.59 (-1.03 to -0.14)	0.012	2001	2023	-0.59 (-1.03 to -0.14)	0.012
	Late-Onset	2001	2014	3.35 (3.01 to 3.68)	<0.001	2001	2023	1.44 (1.19 to 1.70)	<0.001
		2014	2023	-1.24 (-1.70 to -0.78)	<0.001				
Lung	Early-Onset	2001	2006	0.96 (-0.50 to 2.44)	0.180	2001	2023	-3.20 (-3.74 to -2.65)	<0.001
		2006	2017	-5.14 (-5.70 to -4.58)	<0.001				
		2017	2023	-2.99 (-4.50 to -1.47)	<0.001				
	Late-Onset	2001	2006	-0.06 (-0.52 to 0.41)	0.799	2001	2023	-1.26 (-1.47 to -1.06)	<0.001
		2006	2011	-1.91 (-2.56 to -1.26)	<0.001				
		2011	2017	-0.78 (-1.21 to -0.34)	0.002				
		2017	2023	-2.20 (-2.53 to -1.88)	<0.001				
Pancreas	Early-Onset	2001	2023	2.29 (2.15 to 2.44)	<0.001	2001	2023	2.29 (2.15 to 2.44)	<0.001
	Late-Onset	2001	2006	1.03 (0.41 to 1.65)	0.004	2001	2023	0.50 (0.24 to 0.77)	<0.001
		2006	2011	0.12 (-0.71 to 0.96)	0.758				
		2011	2018	0.90 (0.51 to 1.29)	<0.001				
		2018	2023	-0.19 (-0.75 to 0.37)	0.471				

Abbreviations: APC, annual percent change; AAPC, average annual percent change; CI, confidence interval

Supplementary Table S9. APCs and AAPCs among men aged ≥20 years with new cancer cases in the United States from 2001 to 2023 (2020 skipped) by age of onset

Cancer Type	Early-Onset vs. Late-Onset	Joinpoint Segment (Year)		Annual Percent Change (95% CI)	P-value	AAPC Segment (Year)		Average Annual Percent Change (95% CI)	P-value
		Start	End			Start	End		
Colorectal	Early-Onset	2001	2018	1.27 (1.07 to 1.48)	<0.001	2001	2023	2.19 (1.89 to 2.49)	<0.001
		2018	2023	5.35 (4.10 to 6.63)	<0.001				
	Late-Onset	2001	2012	-4.18 (-4.34 to -4.02)	<0.001	2001	2023	-2.83 (-2.94 to -2.72)	<0.001
		2012	2023	-1.47 (-1.65 to -1.30)	<0.001				
Liver	Early-Onset	2001	2005	0.76 (-1.81 to 3.40)	0.533	2001	2023	-3.01 (-3.92 to -2.09)	<0.001
		2005	2012	-3.97 (-5.23 to -2.69)	<0.001				
		2012	2018	-5.64 (-7.61 to -3.63)	<0.001				
		2018	2023	-1.41 (-4.09 to 1.35)	0.283				
	Late-Onset	2001	2009	5.60 (5.03 to 6.17)	<0.001	2001	2023	1.75 (1.46 to 2.05)	<0.001
		2009	2016	2.35 (1.71 to 3.00)	<0.001				
		2016	2023	-3.04 (-3.50 to -2.58)	<0.001				
Lung	Early-Onset	2001	2005	-2.26 (-3.57 to -0.93)	0.003	2001	2023	-4.18 (-4.58 to -3.79)	<0.001
		2005	2017	-5.80 (-6.16 to -5.44)	<0.001				
		2017	2023	-2.16 (-3.28 to -1.03)	0.001				
	Late-Onset	2001	2005	-1.99 (-2.49 to -1.48)	<0.001	2001	2023	-2.90 (-3.06 to -2.74)	<0.001
		2005	2012	-3.08 (-3.33 to -2.83)	<0.001				
		2012	2017	-2.31 (-2.81 to -1.80)	<0.001				
		2017	2023	-3.80 (-4.06 to -3.54)	<0.001				
Pancreas	Early-Onset	2001	2023	0.96 (0.83 to 1.09)	<0.001	2001	2023	0.96 (0.83 to 1.09)	<0.001
	Late-Onset	2001	2013	0.58 (0.49 to 0.66)	<0.001	2001	2023	0.52 (0.40 to 0.65)	<0.001
		2013	2018	1.31 (0.86 to 1.77)	<0.001				
		2018	2023	-0.39 (-0.71 to -0.06)	0.023				
Prostate	Early-Onset	2001	2009	3.68 (2.21 to 5.18)	<0.001	2001	2023	-1.57 (-2.65 to -0.49)	0.004
		2009	2015	-8.36 (-11.16 to -5.48)	<0.001				
		2015	2023	-1.42 (-3.22 to 0.42)	0.119				
	Late-Onset	2001	2008	-1.44 (-3.24 to 0.39)	0.112	2001	2023	-1.49 (-2.53 to -0.45)	0.005
		2008	2014	-6.33 (-9.20 to -3.37)	<0.001				
		2014	2023	1.83 (0.53 to 3.14)	0.009				

Abbreviations: APC, annual percent change; AAPC, average annual percent change; CI, confidence interval

Supplementary Table S10. APCs and AAPCs among individuals aged ≥20 years with new cancer cases in the United States from 2001 to 2023 (2020 skipped) by age group

Cancer Type	Age Category	Annual Percent Change				Average Annual Percent Change			
		Joinpoint Segment (Year)		Estimate (95% CI)	P-value	Study Duration (Year)		Estimate (9% CI)	P-value
		Start	End			Start	End		
Breast (female)	20-39	2001	2010	0.02 (-0.27 to 0.32)	0.881	2001	2023	0.66 (0.51 to 0.80)	<0.001
		2010	2023	1.10 (0.94 to 1.27)	<0.001				
	40-49	2001	2013	0.13 (-0.07 to 0.33)	0.185	2001	2023	0.70 (0.55 to 0.85)	<0.001
		2013	2023	1.40 (1.13 to 1.66)	<0.001				
	50-59	2001	2005	-4.02 (-5.14 to -2.89)	<0.001	2001	2023	-0.45 (-0.75 to -0.15)	0.003
		2005	2011	-0.71 (-1.51 to 0.11)	0.083				
		2011	2023	0.90 (0.68 to 1.12)	<0.001				
	60-69	2001	2005	-2.56 (-3.89 to -1.22)	<0.001	2001	2023	-0.39 (-0.64 to -0.14)	0.003
		2005	2023	0.10 (-0.03 to 0.23)	0.116				
	70+	2001	2005	-3.13 (-4.30 to -1.95)	<0.001	2001	2023	-0.42 (-0.64 to -0.19)	<0.001
		2005	2023	0.19 (0.07 to 0.32)	0.004				
Colorectal	20-39	2001	2023	1.43 (1.33 to 1.54)	<0.001	2001	2023	1.43 (1.33 to 1.54)	<0.001
	40-49	2001	2018	0.97 (0.70 to 1.24)	<0.001	2001	2023	2.27 (1.94 to 2.61)	<0.001
		2018	2023	6.83 (5.50 to 8.17)	<0.001				
	50-59	2001	2005	-0.57 (-1.69 to 0.56)	0.297	2001	2023	-0.08 (-0.36 to 0.21)	0.600
		2005	2011	-1.79 (-2.54 to -1.03)	<0.001				
		2011	2023	0.96 (0.75 to 1.16)	<0.001				
	60-69	2001	2005	-3.16 (-4.17 to -2.15)	<0.001	2001	2023	-2.71 (-2.96 to -2.46)	<0.001
		2005	2011	-5.10 (-5.76 to -4.43)	<0.001				
		2011	2023	-1.34 (-1.51 to -1.17)	<0.001				
	70+	2001	2005	-4.00 (-4.54 to -3.45)	<0.001	2001	2023	-3.77 (-3.91 to -3.63)	<0.001
		2005	2012	-4.92 (-5.23 to -4.62)	<0.001				
		2012	2023	-2.95 (-3.08 to -2.82)	<0.001				
Liver	20-39	2001	2023	0.04 (-0.45 to 0.53)	0.871	2001	2023	0.04 (-0.45 to 0.53)	0.871
	40-49	2001	2005	0.98 (-1.68 to 3.72)	0.437	2001	2023	-3.01 (-4.02 to -1.99)	<0.001
		2005	2013	-3.69 (-4.69 to -2.68)	<0.001				
		2013	2018	-6.81 (-9.81 to -3.71)	<0.001				
		2018	2023	-1.16 (-4.03 to 1.80)	0.404				
	50-59	2001	2007	11.59 (9.79 to 13.42)	<0.001	2001	2023	-0.37 (-1.19 to 0.46)	0.379
		2007	2012	2.63 (0.43 to 4.88)	0.023				
		2012	2017	-5.92 (-7.96 to -3.84)	<0.001				
		2017	2023	-8.97 (-10.36 to -7.56)	<0.001				
	60-69	2001	2006	3.39 (0.71 to 6.14)	0.017	2001	2023	2.92 (2.23 to 3.61)	<0.001
		2006	2016	7.79 (7.00 to 8.59)	<0.001				
		2016	2023	-3.97 (-4.79 to -3.15)	<0.001				
	70+	2001	2007	2.63 (1.83 to 3.42)	<0.001	2001	2023	1.95 (1.53 to 2.37)	<0.001
		2007	2012	1.13 (-0.19 to 2.47)	0.087				
		2012	2018	2.88 (2.02 to 3.74)	<0.001				
		2018	2023	0.85 (0.08 to 1.63)	0.034				
Lung	20-39	2001	2014	-3.03 (-3.52 to -2.54)	<0.001	2001	2023	-1.97 (-2.42 to -1.50)	<0.001
		2014	2023	-0.40 (-1.39 to 0.59)	0.403				
	40-49	2001	2006	-0.61 (-1.69 to 0.47)	0.243	2001	2023	-4.06 (-4.49 to -3.63)	<0.001
		2006	2017	-6.05 (-6.50 to -5.61)	<0.001				
		2017	2023	-3.19 (-4.42 to -1.94)	<0.001				
	50-59	2001	2006	-2.99 (-3.46 to -2.52)	<0.001	2001	2023	-3.05 (-3.20 to -2.90)	<0.001
		2006	2016	-1.43 (-1.61 to -1.25)	<0.001				
		2016	2023	-5.36 (-5.65 to -5.06)	<0.001				

Cancer Type	Age Category	Annual Percent Change				Average Annual Percent Change			
		Joinpoint Segment (Year)		Estimate (95% CI)	P-value	Study Duration (Year)		Estimate (9% CI)	P-value
		Start	End			Start	End		
	60-69	2001	2006	-1.96 (-2.38 to -1.53)	<0.001	2001	2023	-2.15 (-2.28 to -2.01)	<0.001
		2006	2013	-3.89 (-4.19 to -3.59)	<0.001				
		2013	2023	-1.00 (-1.13 to -0.86)	<0.001				
	70+	2001	2006	-0.20 (-0.69 to 0.30)	0.394	2001	2023	-1.80 (-2.03 to -1.57)	<0.001
		2006	2011	-2.06 (-2.76 to -1.35)	<0.001				
		2011	2017	-1.33 (-1.82 to -0.85)	<0.001				
		2017	2023	-3.36 (-3.71 to -3.00)	<0.001				
Pancreas	20-39	2001	2023	3.44 (3.09 to 3.79)	<0.001	2001	2023	3.44 (3.09 to 3.79)	<0.001
	40-49	2001	2023	1.04 (0.93 to 1.14)	<0.001	2001	2023	1.04 (0.93 to 1.14)	<0.001
	50-59	2001	2005	2.07 (0.85 to 3.30)	0.003	2001	2023	0.79 (0.42 to 1.15)	<0.001
		2005	2010	0.37 (-0.69 to 1.44)	0.460				
		2010	2015	1.70 (0.71 to 2.70)	0.003				
		2015	2023	-0.15 (-0.46 to 0.16)	0.305				
	60-69	2001	2011	0.87 (0.69 to 1.06)	<0.001	2001	2023	0.82 (0.66 to 0.98)	<0.001
		2011	2018	1.23 (0.90 to 1.56)	<0.001				
		2018	2023	0.14 (-0.33 to 0.61)	0.548				
	70+	2001	2005	0.84 (-0.20 to 1.89)	0.105	2001	2023	0.35 (0.04 to 0.66)	0.025
		2005	2013	0.10 (-0.29 to 0.48)	0.598				
		2013	2018	1.26 (0.34 to 2.18)	0.011				
		2018	2023	-0.52 (-1.15 to 0.12)	0.103				
Prostate	20-39	2001	2009	5.96 (2.85 to 9.16)	<0.001	2001	2023	-2.14 (-4.60 to 0.38)	0.095
		2009	2014	-12.06 (-20.09 to -3.21)	0.012				
		2014	2023	-3.24 (-6.38 to 0.00)	0.050				
	40-49	2001	2009	3.63 (2.16 to 5.12)	<0.001	2001	2023	-1.56 (-2.63 to -0.48)	0.005
		2009	2015	-8.30 (-11.09 to -5.42)	<0.001				
		2015	2023	-1.40 (-3.20 to 0.43)	0.123				
	50-59	2001	2009	0.42 (-1.34 to 2.22)	0.619	2001	2023	-1.29 (-2.50 to -0.07)	0.039
		2009	2014	-6.62 (-10.57 to -2.49)	0.004				
		2014	2023	0.26 (-1.35 to 1.90)	0.738				
	60-69	2001	2009	-0.85 (-2.63 to 0.97)	0.333	2001	2023	-1.21 (-2.43 to 0.02)	0.054
		2009	2014	-6.57 (-10.72 to -2.23)	0.006				
		2014	2023	1.57 (0.14 to 3.01)	0.033				
	70+	2001	2008	-2.72 (-4.24 to -1.17)	0.002	2001	2023	-1.81 (-2.87 to -0.75)	<0.001
		2008	2013	-8.31 (-12.15 to -4.31)	<0.001				
		2013	2023	2.26 (1.26 to 3.28)	<0.001				

Abbreviations: APC, annual percent change; AAPC, average annual percent change; CI, confidence interval

Supplementary Table S11. APCs and AAPCs Among Women Aged ≥20 years with New Cancer Cases in the United States from 2001 to 2023 (2020 Skipped) by Age of Onset and Stage at Diagnosis

Cancer Type	Age at Onset	Stage at Diagnosis	Annual Percent Change				Average Annual Percent Change			
			Joinpoint Segment (Year)		Estimate (95% CI)	P-value	Study Duration (Year)		Estimate (95% CI)	P-value
			Start	End			Start	End		
Breast (female)	Early-Onset	Early	2001	2012	0.46 (0.18 to 0.75)	0.003	2001	2023	1.10 (0.91 to 1.28)	<0.001
			2012	2023	1.73 (1.45 to 2.02)	<0.001				
		Late	2001	2005	0.92 (-0.25 to 2.11)	0.113	2001	2023	0.58 (0.31 to 0.84)	<0.001
			2005	2016	-0.02 (-0.29 to 0.25)	0.879				
			2016	2023	1.32 (0.85 to 1.79)	<0.001				
		Unstaged	2001	2007	-10.69 (-13.34 to -7.95)	<0.001	2001	2023	-4.20 (-5.75 to -2.63)	<0.001
			2007	2018	-0.04 (-1.61 to 1.55)	0.958				
			2018	2023	-5.10 (-10.66 to 0.80)	0.084				
	Late-Onset	Early	2001	2005	-4.04 (-5.29 to -2.76)	<0.001	2001	2023	0.02 (-0.23 to 0.26)	0.885
			2005	2023	0.94 (0.81 to 1.07)	<0.001				
		Late	2001	2023	-0.88 (-1.00 to -0.77)	<0.001	2001	2023	-0.88 (-1.00 to -0.77)	<0.001
		Unstaged	2001	2007	-9.86 (-11.47 to -8.22)	<0.001	2001	2023	-4.33 (-5.19 to -3.47)	<0.001
			2007	2018	-1.29 (-2.17 to -0.40)	0.008				
			2018	2023	-4.10 (-7.08 to -1.02)	0.013				
Colorectal	Early-Onset	Early	2001	2009	2.35 (0.49 to 4.24)	0.017	2001	2023	2.38 (1.18 to 3.59)	<0.001
			2009	2018	-2.32 (-4.11 to -0.49)	0.017				
			2018	2023	11.45 (7.57 to 15.48)	<0.001				
		Late	2001	2011	0.77 (0.33 to 1.22)	0.002	2001	2023	1.89 (1.64 to 2.14)	<0.001
			2011	2023	2.83 (2.51 to 3.15)	<0.001				
		Unstaged	2001	2008	-4.43 (-6.69 to -2.11)	<0.001	2001	2023	-0.48 (-1.33 to 0.38)	0.273
			2008	2023	1.42 (0.63 to 2.21)	0.001				
	Late-Onset	Early	2001	2005	0.94 (-1.27 to 3.19)	0.382	2001	2023	-3.00 (-3.58 to -2.41)	<0.001
			2005	2018	-4.51 (-4.95 to -4.07)	<0.001				
			2018	2023	-2.11 (-4.03 to -0.14)	0.038				
		Late	2001	2005	-5.09 (-6.21 to -3.95)	<0.001	2001	2023	-2.36 (-2.65 to -2.07)	<0.001
			2005	2012	-3.55 (-4.17 to -2.93)	<0.001				
			2012	2023	-0.57 (-0.82 to -0.32)	<0.001				
		Unstaged	2001	2007	-7.06 (-7.74 to -6.38)	<0.001	2001	2023	-3.73 (-4.26 to -3.21)	<0.001
			2007	2012	-4.07 (-5.63 to -2.49)	<0.001				
			2012	2017	0.93 (-0.71 to 2.61)	0.239				
			2017	2023	-3.86 (-4.70 to -3.02)	<0.001				
Liver	Early-Onset	Early	2001	2023	1.11 (0.45 to 1.77)	0.002	2001	2023	1.11 (0.45 to 1.77)	0.002
		Late	2001	2013	-0.26 (-1.37 to 0.85)	0.617	2001	2023	-0.61 (-2.71 to 1.54)	0.574
			2013	2018	-7.04 (-14.04 to 0.52)	0.065				
			2018	2023	5.38 (-0.87 to 12.03)	0.087				
		Unstaged	2001	2023	-3.00 (-3.66 to -2.32)	<0.001	2001	2023	-3.00 (-3.66 to -2.32)	<0.001
	Late-Onset	Early	2001	2007	10.09 (7.85 to 12.38)	<0.001	2001	2023	3.70 (2.99 to 4.41)	<0.001
			2007	2016	3.50 (2.54 to 4.47)	<0.001				
			2016	2023	-1.25 (-2.23 to -0.26)	0.017				
		Late	2001	2013	3.78 (3.21 to 4.35)	<0.001	2001	2023	1.70 (0.95 to 2.46)	<0.001
			2013	2018	-3.02 (-5.56 to -0.41)	0.027				
			2018	2023	1.60 (-0.39 to 3.63)	0.107				
		Unstaged	2001	2006	-4.19 (-7.48 to -0.78)	0.020	2001	2023	-2.20 (-3.32 to -1.06)	<0.001
			2006	2018	0.57 (-0.40 to 1.54)	0.229				
			2018	2023	-6.61 (-9.97 to -3.13)	0.001				

Cancer Type	Age at Onset	Stage at Diagnosis	Annual Percent Change				Average Annual Percent Change			
			Joinpoint Segment (Year)		Estimate (95% CI)	P-value	Study Duration (Year)		Estimate (95% CI)	P-value
			Start	End			Start	End		
Lung	Early-Onset	Early	2001	2008	-0.15 (-2.29 to 2.03)	0.882	2001	2023	-0.92 (-2.21 to 0.39)	0.168
			2008	2014	-4.67 (-8.25 to -0.94)	0.018				
			2014	2023	1.06 (-0.67 to 2.81)	0.211				
		Late	2001	2005	2.74 (1.29 to 4.21)	0.002	2001	2023	-3.48 (-3.99 to -2.95)	<0.001
			2005	2010	-3.88 (-5.22 to -2.51)	<0.001				
			2010	2018	-6.20 (-6.86 to -5.54)	<0.001				
			2018	2023	-3.47 (-5.00 to -1.92)	<0.001				
		Unstaged	2001	2013	-8.41 (-9.43 to -7.38)	<0.001	2001	2023	-6.44 (-7.50 to -5.37)	<0.001
			2013	2023	-4.03 (-6.25 to -1.76)	0.002				
	Late-Onset	Early	2001	2013	-0.58 (-1.21 to 0.05)	0.069	2001	2023	1.13 (0.39 to 1.88)	0.003
			2013	2018	5.82 (3.01 to 8.70)	<0.001				
			2018	2023	0.69 (-1.07 to 2.48)	0.416				
		Late	2001	2005	1.90 (0.64 to 3.18)	0.006	2001	2023	-1.50 (-1.78 to -1.21)	<0.001
			2005	2014	-1.29 (-1.69 to -0.88)	<0.001				
			2014	2023	-3.17 (-3.51 to -2.84)	<0.001				
		Unstaged	2001	2005	-8.08 (-10.94 to -5.12)	<0.001	2001	2023	-5.06 (-6.22 to -3.89)	<0.001
			2005	2013	-4.85 (-6.28 to -3.41)	<0.001				
			2013	2018	-0.94 (-4.71 to 2.97)	0.600				
			2018	2023	-6.95 (-9.76 to -4.06)	<0.001				
Pancreas	Early-Onset	Early	2001	2010	6.69 (5.33 to 8.06)	<0.001	2001	2023	8.43 (7.56 to 9.30)	<0.001
			2010	2016	13.85 (11.24 to 16.52)	<0.001				
			2016	2023	6.18 (5.13 to 7.23)	<0.001				
		Late	2001	2009	2.02 (1.16 to 2.88)	<0.001	2001	2023	0.78 (0.43 to 1.14)	<0.001
			2009	2023	0.08 (-0.26 to 0.43)	0.614				
		Unstaged	2001	2012	-3.78 (-5.75 to -1.78)	0.001	2001	2023	-1.20 (-2.63 to 0.24)	0.103
			2012	2023	1.45 (-0.91 to 3.86)	0.214				
	Late-Onset	Early	2001	2013	2.34 (1.29 to 3.39)	<0.001	2001	2023	4.15 (3.17 to 5.14)	<0.001
			2013	2018	9.74 (6.19 to 13.41)	<0.001				
			2018	2023	3.08 (1.06 to 5.13)	0.005				
		Late	2001	2006	2.60 (1.63 to 3.58)	<0.001	2001	2023	0.80 (0.52 to 1.09)	<0.001
			2006	2015	0.80 (0.38 to 1.22)	0.001				
			2015	2023	-0.30 (-0.67 to 0.08)	0.111				
		Unstaged	2001	2013	-4.05 (-4.59 to -3.50)	<0.001	2001	2023	-3.60 (-4.52 to -2.66)	<0.001
			2013	2018	0.05 (-3.38 to 3.60)	0.976				
			2018	2023	-6.05 (-8.57 to -3.46)	<0.001				

Abbreviations: APC, annual percent change; AAPC, average annual percent change; CI, confidence interval

Supplementary Table S12. APCs and AAPCs among men aged ≥20 years with new cancer cases in the United States from 2001 to 2023 (2020 skipped) by age of onset and stage at diagnosis

Cancer Type	Age at Onset	Stage at Diagnosis	Annual Percent Change				Average Annual Percent Change (95% CI)			
			Joinpoint Segment (Year)		Estimate (95% CI)	P-value	Study Duration (Year)		Estimate (95% CI)	P-value
			Start	End			Start	End		
Colorectal	Early-Onset	Early	2001	2007	3.24 (0.66 to 5.89)	0.017	2001	2023	2.22 (1.11 to 3.33)	<0.001
			2007	2018	-1.78 (-2.92 to -0.63)	0.005				
			2018	2023	10.27 (6.57 to 14.10)	<0.001				
		Late	2001	2012	1.63 (1.40 to 1.86)	<0.001	2001	2023	2.45 (2.31 to 2.60)	<0.001
			2012	2023	3.28 (3.07 to 3.49)	<0.001				
		Unstaged	2001	2012	-2.45 (-4.04 to -0.83)	0.005	2001	2023	-0.03 (-1.12 to 1.07)	0.954
			2012	2023	2.45 (0.72 to 4.20)	0.008				
	Late-Onset	Early	2001	2005	-0.42 (-2.93 to 2.16)	0.730	2001	2023	-3.40 (-4.06 to -2.74)	<0.001
			2005	2018	-4.85 (-5.33 to -4.36)	<0.001				
			2018	2023	-1.96 (-4.09 to 0.22)	0.074				
		Late	2001	2007	-5.22 (-5.96 to -4.48)	<0.001	2001	2023	-2.22 (-2.61 to -1.83)	<0.001
			2007	2012	-2.97 (-4.46 to -1.46)	<0.001				
			2012	2023	-0.19 (-0.50 to 0.12)	0.205				
		Unstaged	2001	2007	-7.32 (-7.85 to -6.78)	<0.001	2001	2023	-3.87 (-4.27 to -3.47)	<0.001
			2007	2012	-3.96 (-5.17 to -2.75)	<0.001				
			2012	2017	0.63 (-0.60 to 1.88)	0.287				
			2017	2023	-3.95 (-4.56 to -3.33)	<0.001				
Liver	Early-Onset	Early	2001	2005	5.70 (1.07 to 10.54)	0.018	2001	2023	-1.80 (-2.61 to -0.99)	<0.001
			2005	2023	-3.40 (-3.82 to -2.97)	<0.001				
		Late	2001	2005	0.85 (-3.49 to 5.38)	0.680	2001	2023	-2.86 (-4.52 to -1.17)	<0.001
			2005	2013	-3.32 (-5.01 to -1.60)	0.001				
			2013	2018	-8.55 (-13.38 to -3.46)	0.004				
			2018	2023	0.92 (-3.79 to 5.85)	0.682				
		Unstaged	2001	2023	-6.51 (-7.10 to -5.92)	<0.001	2001	2023	-6.51 (-7.10 to -5.92)	<0.001
	Late-Onset	Early	2001	2007	11.17 (9.48 to 12.89)	<0.001	2001	2023	3.47 (2.96 to 3.99)	<0.001
			2007	2017	3.57 (3.03 to 4.12)	<0.001				
			2017	2023	-3.86 (-4.75 to -2.96)	<0.001				
		Late	2001	2012	5.59 (4.63 to 6.56)	<0.001	2001	2023	2.04 (1.50 to 2.59)	<0.001
			2012	2023	-1.39 (-2.07 to -0.70)	<0.001				
		Unstaged	2001	2005	-5.01 (-7.95 to -1.98)	0.004	2001	2023	-2.38 (-3.30 to -1.46)	<0.001
			2005	2010	2.21 (-0.71 to 5.22)	0.126				
			2010	2018	0.59 (-0.41 to 1.61)	0.220				
			2018	2023	-9.19 (-11.10 to -7.24)	<0.001				
Lung	Early-Onset	Early	2001	2013	-4.78 (-5.53 to -4.02)	<0.001	2001	2023	-3.03 (-3.69 to -2.37)	<0.001
			2013	2023	-0.89 (-2.18 to 0.41)	0.166				
		Late	2001	2005	-1.08 (-2.46 to 0.31)	0.117	2001	2023	-4.08 (-4.50 to -3.65)	<0.001
			2005	2018	-5.83 (-6.13 to -5.53)	<0.001				
			2018	2023	-1.79 (-3.39 to -0.17)	0.033				
		Unstaged	2001	2014	-9.36 (-10.13 to -8.59)	<0.001	2001	2023	-7.07 (-8.06 to -6.07)	<0.001
			2014	2023	-3.66 (-6.07 to -1.18)	0.006				
	Late-Onset	Early	2001	2013	-2.92 (-3.46 to -2.37)	<0.001	2001	2023	-0.69 (-1.45 to 0.08)	0.078
			2013	2018	5.05 (2.09 to 8.10)	0.002				
			2018	2023	-0.86 (-2.71 to 1.04)	0.347				
		Late	2001	2005	-0.51 (-1.40 to 0.40)	0.247	2001	2023	-3.05 (-3.26 to -2.84)	<0.001
			2005	2014	-2.71 (-3.01 to -2.41)	<0.001				

Cancer Type	Age at Onset	Stage at Diagnosis	Annual Percent Change				Average Annual Percent Change (95% CI)			
			Joinpoint Segment (Year)		Estimate (95% CI)	P-value	Study Duration (Year)		Estimate (95% CI)	P-value
			Start	End			Start	End		
			2014	2023	-4.49 (-4.75 to -4.23)	<0.001				
		Unstaged	2001	2005	-8.77 (-11.53 to -5.92)	<0.001	2001	2023	-5.85 (-6.96 to -4.73)	<0.001
			2005	2013	-5.12 (-6.48 to -3.75)	<0.001				
			2013	2018	-2.54 (-6.11 to 1.17)	0.158				
			2018	2023	-7.89 (-10.63 to -5.06)	<0.001				
Pancreas	Early-Onset	Early	2001	2008	3.08 (-1.11 to 7.46)	0.139	2001	2023	6.33 (4.49 to 8.21)	<0.001
			2008	2018	10.51 (8.26 to 12.82)	<0.001				
			2018	2023	2.82 (-1.69 to 7.53)	0.205				
		Late	2001	2006	1.97 (0.47 to 3.49)	0.013	2001	2023	0.53 (0.18 to 0.88)	0.003
			2006	2023	0.11 (-0.10 to 0.32)	0.288				
		Unstaged	2001	2023	-2.43 (-2.96 to -1.90)	<0.001	2001	2023	-2.43 (-2.96 to -1.90)	<0.001
	Late-Onset	Early	2001	2013	2.58 (1.70 to 3.47)	<0.001	2001	2023	4.71 (3.79 to 5.64)	<0.001
			2013	2018	10.95 (7.40 to 14.62)	<0.001				
			2018	2023	3.82 (1.91 to 5.76)	<0.001				
		Late	2001	2005	2.30 (1.58 to 3.02)	<0.001	2001	2023	0.71 (0.49 to 0.92)	<0.001
			2005	2010	0.74 (0.10 to 1.38)	0.027				
			2010	2015	1.21 (0.63 to 1.80)	<0.001				
			2015	2023	-0.42 (-0.59 to -0.25)	<0.001				
		Unstaged	2001	2013	-3.41 (-3.92 to -2.90)	<0.001	2001	2023	-3.09 (-3.93 to -2.25)	<0.001
			2013	2018	-0.06 (-3.17 to 3.14)	0.966				
			2018	2023	-5.29 (-7.58 to -2.95)	<0.001				
Prostate	Early-Onset	Early	2001	2009	4.35 (2.77 to 5.96)	<0.001	2001	2023	-1.85 (-3.01 to -0.68)	0.002
			2009	2015	-9.32 (-12.29 to -6.25)	<0.001				
			2015	2023	-2.04 (-4.01 to -0.02)	0.048				
		Late	2001	2008	3.98 (2.79 to 5.19)	<0.001	2001	2023	-0.16 (-1.23 to 0.93)	0.778
			2008	2013	-5.76 (-8.46 to -2.99)	<0.001				
			2013	2018	-2.47 (-5.65 to 0.81)	0.124				
			2018	2023	2.31 (-0.32 to 5.01)	0.079				
		Unstaged	2001	2005	-10.69 (-19.34 to -1.11)	0.032	2001	2023	-2.81 (-4.70 to -0.88)	0.005
			2005	2023	-0.97 (-2.20 to 0.28)	0.121				
	Late-Onset	Early	2001	2008	-0.65 (-2.67 to 1.41)	0.505	2001	2023	-1.95 (-3.12 to -0.76)	0.001
			2008	2014	-8.29 (-11.46 to -5.01)	<0.001				
			2014	2023	1.48 (-0.05 to 3.04)	0.057				
		Late	2001	2013	-0.62 (-1.11 to -0.13)	0.016	2001	2023	1.50 (1.17 to 1.84)	<0.001
			2013	2023	4.12 (3.57 to 4.66)	<0.001				
		Unstaged	2001	2005	-13.18 (-19.20 to -6.70)	<0.001	2001	2023	-3.85 (-5.12 to -2.55)	<0.001
			2005	2023	-1.64 (-2.35 to -0.92)	<0.001				

Abbreviations: APC, annual percent change; AAPC, average annual percent change; CI, confidence interval
